# High-dimensional single-cell phenotyping unveils persistent differences in immune cell profiles between severe and moderate seasonal influenza

**DOI:** 10.3389/fimmu.2025.1576861

**Published:** 2025-07-22

**Authors:** Johanna Bodin, Gro Tunheim, Anja B. Kristoffersen, Tove K. Herstad, Eleonora Vianello, Mariëlle C. Haks, Suzanne van Veen, Torgun Wæhre, Anne-Marte B. Kran, Sarah L. Lartey, Fan Zhou, Rebecca J. Cox, Tom H. M. Ottenhoff, Anne M. Dyrhol-Riise, Unni C. Nygaard, Fredrik Oftung, Siri Mjaaland

**Affiliations:** ^1^ Section of Immunology, Department of Method Development and Analytics, Norwegian Institute of Public Health, Oslo, Norway; ^2^ Department of Infectious Diseases, Leiden University Medical Center, Leiden, Netherlands; ^3^ Department of Infectious Diseases, Oslo University Hospital, Oslo, Norway; ^4^ Division of Infection Control, Department of Infectious Disease Registries, Norwegian Institute of Public Health, Oslo, Norway; ^5^ Department of Microbiology, Oslo University Hospital, Oslo, Norway; ^6^ Influenza Centre, Department of Clinical Science, University of Bergen, Bergen, Norway; ^7^ Department of Microbiology, Haukeland University Hospital, Bergen, Norway; ^8^ Institute of Clinical Medicine, University of Oslo, Oslo, Norway

**Keywords:** influenza, hospitalization, disease severity, mass cytometry, immune profiling, biomarkers

## Abstract

**Background:**

Influenza viruses with pandemic potential and possible burden of post-viral sequelae are a global concern. To prepare for future pandemics and the development of improved vaccines, it is vital to identify the immunological changes underlying influenza disease severity.

**Methods:**

We combined unsupervised high-dimensional single-cell mass cytometry with gene expression analyses, plasma CXCL13 measurements, and antigen-specific immune cell assays to characterize the immune profiles of hospitalized patients with severe and moderate seasonal influenza disease during active infection and at 6-month follow-up. We used age-matched healthy donors as controls.

**Results:**

Severe disease was associated with a distinct immune profile, including lower frequencies of ICOS^+^ mucosal-associated invariant T (MAIT) cells, and CXCR5^+^ memory B and CD4^+^CXCR5^+^CD95^+^ICOS^+^ and CD8^+^CXCR3^+^CD95^+^PD-1^+^TIGIT^+^ memory T cells, as well as lower CD4 gene expression. Higher frequencies of CD16^+^CD161^+^ NK cells, CD169^+^ monocytes, CD123^+/−^ dendritic cells, and CD38^high^ plasma cells and high CXCL13 plasma levels were also associated with severe disease. Alterations in immune cell subpopulations persisted at convalescence for the severely ill patients only.

**Conclusions:**

Our results indicated a reduction in regulatory MAIT cells and memory T and B cells and an increase in the inhibitory subpopulations of monocytes and NK cells in severe influenza that persisted at convalescence. These immune cell alterations were associated with higher age and the presence of several underlying conditions that may contribute to frailty. This study illustrates the power and sensitivity of high-dimensional single-cell analyses in identifying potential cellular biomarkers for disease severity after influenza infection.

## Introduction

Influenza is ranked among the greatest global health threats in the 21st century (1). This is particularly relevant in light of the current outbreak of H5N1 in poultry and dairy cows, which is a major concern due to its pandemic potential. The estimated overall influenza-related excess mortality in Norway prior to the COVID-19 pandemic reached 910 deaths per season, accounting for 2.08% of all deaths, and worldwide, the number of deaths exceeded 600,000 (2, 3). The average annual hospitalization rate due to influenza in Norway between 2008 and 2017, overlapping the period when the sampling was performed (2014–2017), was 48 per 100,000 (including the 2009–2010 pandemic outbreak) (4). Risk factors associated with severe outcomes include age over 65 years and underlying risk factors such as immunodeficiency, obesity, cardiovascular diseases, neuromuscular diseases, diabetes, and pregnancy (5, 6). Long-term effects after influenza infection are similar to those after COVID-19 conditions, but less severe, except for pulmonary-related outcomes like cough and shortness of breath (7). Understanding immunological alterations in patients with severe influenza is crucial in preparing for future pandemics, preventing post-viral sequelae, and developing better vaccines.

The progression and outcome of influenza infection are determined by a complex interplay between innate and adaptive immune responses. Protection against influenza infection has been associated with various factors, including the presence of influenza-specific serum antibodies, polyfunctional B and T cells, particularly IFNγ-producing CD4^+^ T cells, memory CD8^+^ T cells, and CD38^+^ plasmacytoid dendritic cells (pDCs) (8–15). Additionally, innate immune cells such as NK cells, monocytes, and dendritic cells (DCs) contribute to viral containment and immune cell recruitment through the secretion of cytokines, interferons, and chemokines (11–13). Genes related to antimicrobial responses and neutrophil activity are also reported to be upregulated in whole blood in response to influenza infection (16–18).

Severe influenza disease is associated with impaired control of viral replication, defective interferon responses, or deficiencies in cell-mediated immunity (9, 19, 20). Severe and life-threatening influenza is characterized by lymphopenia, with a decrease in circulating CD4^+^ and CD8^+^ T cells, γδ T cells, and mucosal-associated invariant T (MAIT) cells, along with an excessive release of proinflammatory cytokines and chemokines (cytokine storm) (19–22). Prolonged inflammation with increased monocyte numbers and elevated cytokine levels is also observed during severe influenza disease (23–25). While there is more limited information on NK cell frequencies and function in severe influenza, both increased activation and decreased frequencies of specific subpopulations have been reported (12, 13), making the role of NK cells in disease outcome still a subject of debate.

In this study, we aimed to identify an immune cell profile associated with severe influenza disease using high-dimensional single-cell immune profiling. Such profiling, linked to clinical outcome, could reveal biomarkers to guide improved treatment and vaccine development targeting beneficial immune responses. By applying mass cytometry (CyTOF) and data-driven analyses, we characterized and compared a variety of immune cell subpopulations in severely and moderately ill influenza patients with polymerase chain reaction (PCR)-confirmed seasonal influenza during both the acute and convalescent phases (26). We included healthy individuals as controls. To support this exploratory approach, we conducted differential gene expression analysis, measured plasma chemokine CXCL13 levels [as a measure of T follicular helper (T_fh_) and B-cell activity prior to antibody induction (27)], and assessed influenza-specific antibody and T-cell responses.

## Methods

### Study population and sample collection

Patients aged ≥18 years admitted to Oslo University Hospital during the four consecutive influenza seasons between 2014/2015 and 2017/2018 were included. Influenza was confirmed by an in-house influenza virus A/B RNA real-time PCR, and Ct values were used as a proxy for viral load to categorize the samples as high or moderate levels of influenza virus (Ct < 28 and Ct = 28.1–33, respectively).

**Figure 1:**
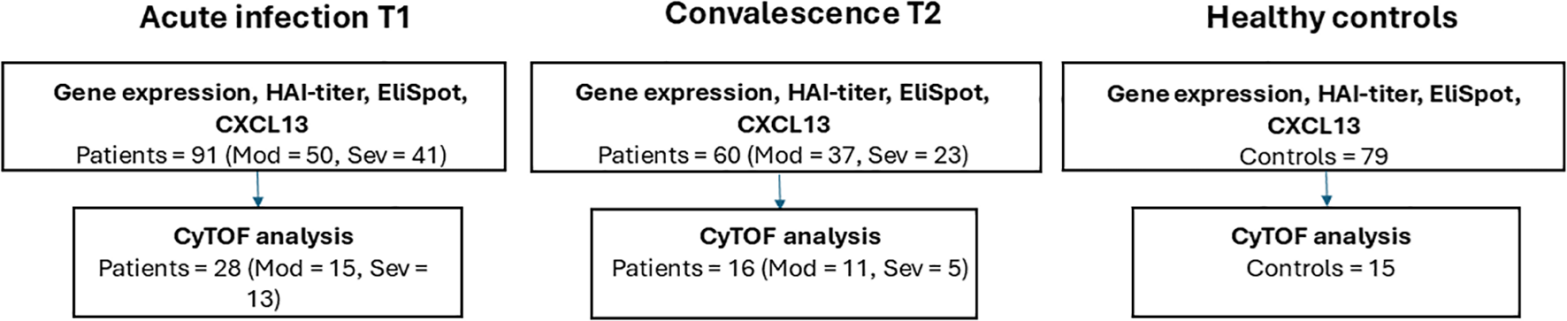
Flowchart of all study participants and analyses conducted. T1, at infection; T2, at convalescence.

Blood samples were collected at the time of hospitalization [i.e., in the acute phase (T1), 0–3 days after admission] and/or in the convalescent phase [5–11 months later (T2)]. Samples from 27 patients with influenza A/H3N2 at acute infection (seasons 1–4) and 16 paired samples at convalescence (seasons 1, 3, and 4, 5–7 months after infection) were analyzed by CyTOF (**Figure 1**). In the other immune analyses, 91 patients at T1 and 60 patients during convalescence (57 paired samples) admitted during the last three seasons were included since RNA was only collected in these seasons (**Figure 1**).

Healthy controls (N = 79), i.e., volunteer donors with no clinical signs of infection at the time of sampling, gave blood samples in the early autumn of 2016 (prior to the start of the influenza season). All controls were included in the immunological and gene expression analyses, and 15 individuals were included in the CyTOF analysis.

Written informed consent was obtained from all participants before inclusion, and the study was approved by the Regional Committee for Medical and Health Research Ethics in South-Eastern Norway (REK number 2013/2033).

### Disease severity definition

Severe disease was retrospectively defined as having at least two of the following criteria: receiving oxygen supplementation, pneumonia at admission, or staying 5 days or longer in hospital. Patients having zero or one criterion were defined as moderately ill. For the CyTOF analysis, all of the severely ill patients were selected on the requirement of oxygen supplementation, together with either pneumonia or staying 5 days or longer at the hospital, whereas none of the moderately ill patients selected in this analysis required oxygen, which improved the clinical separation of the two groups.

### Blood samples

Blood samples for the isolation of Peripheral Blood Mononuclear Cell (PBMCs) and plasma were collected using CPT sodium citrate tubes (BD Vacutainer^®^ CPT™, 362782, Becton, Dickinson and Company, Franklin Lakes, New Jersey, USA). Whole blood was collected using PAXgene Blood RNA tubes (, #762165, PreAnalytiX GmbH, Hombrechtikon, Switzerland). PAXgene samples were collected during seasons 2–4. For the controls, blood samples were collected in Acid citric dextrose (ACD) BD Vacutainer tubes (#364606, Becton, Dickinson and Company, Franklin Lakes, New Jersey, USA), and PBMCs were isolated in SepMate tubes with a dilution of the blood 1:1 in 0.9% NaCl before overlaying the Lymphoprep solution. Plasma was removed from the supernatant after centrifugation. The dilution factor for plasma after PBMC isolation in SepMate tubes was calculated to be 3.53 by comparing levels of vitamins in plasma obtained from healthy blood donors using SepMate and ACD tubes (28).

### CMV ELISA

Cytomegalovirus (CMV) ELISA was performed using CMV-IgG ELISA (EI 2570–9601 G) from Euroimmun (Lübeck, Germany) according to the manufacturer’s instructions. Ten microliters of plasma was used in the analysis, and the plate was read using a Biotek EL808 plate reader at 450/630 nm with the Gen5 software (Thermo Fisher Scientific, Waltham, MA, USA). Anti-CMV IgG ≥ 22 RU/mL was considered positive.

### Mass cytometry antibody staining and acquisition

CyTOF analysis was performed on PBMCs from a subgroup of patients with influenza A/H3N2 infection (12 severely and 15 moderately ill), with follow-up samples at convalescence (five from the severe and 11 from the moderately ill group) and 15 healthy controls. PBMCs were thawed, counted, and split into two tubes of 2 and 3 million live cells before resting overnight at 37°C with 5% CO_2_. Unspecific staining was blocked with Human TruStain Fc (cat 422302, BioLegend, San Diego, CA, USA). The tube of 2 million cells was stained for phenotypic characterization with antibodies against surface markers (**Supplementary Table 2** and annotation of subpopulations according to **Supplementary Table 3**). The cells were stained with surface markers using different staining conditions with washes between the staining steps (**Supplementary Table 6**) and cisplatin (ID™ Cisplatin-194Pt, Fluidigm, San Francisco, CA, USA) for live cell identification, followed by fixation and staining with intercalator Cell-ID Intercalator-Ir (201192B, Fluidigm, San Francisco, CA, USA) for DNA detection. The tube of 3 million cells was stimulated for 4 hours with 1X eBioscience™ Cell Stimulation Cocktail [phorbol 12-myristate 13-acetate (PMA), ionomycin, brefeldin A, and monensin (00-4975-93, eBioscience/Thermo Fisher Scientific, Waltham, MA, USA)]; for characterization of functional cell populations (to investigate how the severity of influenza disease affects the overall immune cell distribution), the PBMCs were stimulated with PMA+ionomycin for maximal activation to identify functional alterations in the overall immune cell population associated with severe influenza infection. The stimulated cells were fixed and permeabilized after surface staining and kept in methanol at −80°C until intracellular staining on the same day as acquisition (staining groups and conditions in **Supplementary Tables 5** and **6**). After two washes and resuspension in water, all samples were analyzed using a Helios CyTOF mass cytometer (Fluidigm, San Francisco, CA, USA). All centrifugation steps were performed at 300 × *g* before fixation and 800 × *g* after fixation and permeabilization.

### CyTOF data analysis

The data files were normalized using EQ Four Element Calibration Beads (#201078, Fluidigm, San Francisco, CA, USA) and spiked into all samples at a 1:10 ratio, followed by algorithmic processing, and then data files were exported for clean-up and analysis in R (version 4.2.2). All codes can be found on GitHub (https://github.com/folkehelseinstituttet/immunological_profiling_severe_influenza).

Clean-up gating was conducted semi-automatically with an in-house routine based on Gaussian parameters following the approach described in Fluidigm Technical note PN 400248 B1. All gates were manually checked and adjusted if needed. Supervised analysis was conducted by gating all cells into positive or negative for marker expression. For some markers, cells were additionally gated as having dim/low or bright/high expression. The gating was conducted semi-automatically using density plots and manually checked and adjusted if needed. Based on this categorization of the expression of each marker, the major cell types (annotated according to **Supplementary Table 3**) were found as a combination of marker expression according to **Supplementary Table 4**, and the cell count of each cell type in each sample was obtained. Negative binomial regression was used to analyze the association between the count of each cell type and the covariates: disease status–timepoint [ST1, severe influenza (S) at active infection (T1); ST2, severe influenza at convalescence (T2); MT1, moderate (M) influenza at infection; MT2, moderate influenza at convalescence; Ctrl, healthy influenza PCR-negative age-matched controls sampled outside the flu season], age, and sex, with ST1 as baseline. To allow the model to represent frequencies rather than counts, the logarithm of the total number of cells per sample was used as an offset. For each cell type, all additive models involving a combination of disease status–timepoint, age, and sex were run in addition to a model with no covariates. Models with additive covariates were used to assume that age and sex impact the cell population size similarly, independent of disease status–timepoint. The best model was found as the simplest model with an Akaike information criterion (AIC) value less than the minimum AIC value + 2. The p-value describing the difference between ST1 and MT1 for each cluster was collected. If a model without disease status–timepoint was found to have the lowest AIC value for a cluster, the p-value from the full model including status–timepoint, age, and sex was collected. Based on all collected p-values, an adjusted p-value was found by false discovery rate (FDR) using the Benjamini–Hochberg method, and cell types and cell populations with adjusted p-value less than 0.05 were reported.

### CyTOF clustering and identification of cells

Unsupervised analyses were conducted by clustering using flow self-organizing map (FlowSOM) with 25,000 cells randomly chosen from each sample. A total of 196 nodes with a predefined number of granularities—10, 20, 30, 40, 50, and 60 clusters—were evaluated, and clusters where the frequency per sample was less than 99% correlated from the different meta-clusters were kept for further evaluation. Clusters where the frequency per sample was correlated between 99% and 99.999% were also kept if the size of the new cluster was <95% of the correlated cluster. Negative binomial regression analysis was performed on the obtained clusters in the same way as for the supervised analysis. The FlowSOM analysis was repeated four times, and the results were compared. Only clusters found in at least two runs were reported as significant. Clusters from different runs that were more than 95% correlated with another cluster were considered similar. Marker expression for the different clusters were visualized using marker plots, where the signal of each marker was plotted as lines with vertical bars representing 5, 10, 25, 75, 90, and 95 quantiles; circles representing median signal; and vertical dotted lines indicating negative/positive signal and for some markers also low/high signals based on manual gating. The marker plots were used to identify the tentative cell type and characteristics of each cluster. To create two-dimensional representations of the dataset, each sample was randomly down-sampled to retain 3,000 cells to form a dataset for the t-SNE algorithm, creating a t-distributed stochastic neighbor embedding (t-SNE) map. The cell populations and the significant clusters were colored in the t-SNE plot based on the results from the supervised gating and significant clusters from unsupervised clustering, respectively.

To verify the findings from the unsupervised clustering, the markers most clearly defining a significant cluster were identified and used for semi-automatic gating of all collected cells per sample (no down-sampling). The number of cells per participant in these gated populations was obtained and analyzed with negative binomial regression to see if they were significantly different between ST1 and MT1. Further, a set of two markers per population was applied to identify cell populations that differed in severity after manual gating. An additional statistical analysis was conducted with ST1, MT1, ST2, and MT2 as reference in the negative binomial regression to identify clusters that were significantly different between any of the study groups at T1 or T2, i.e., ST1–MT1, ST1–Ctrl, MT1–Ctrl, ST2–MT2, ST2–Ctrl, and MT2–Ctrl. The incidence rate ratios (IRRs) calculated in the negative binomial regression were obtained for all the significant clusters for each set of comparisons. All significant clusters were characterized based on marker expression using the marker plots.

### dcRT-MLPA transcription and differential gene expression analysis

A dual-color Reverse Transcriptase Multiplex Ligation-dependent Probe Amplification (dcRT-MLPA) was performed on isolated RNA (using Qiagen RNA isolation kit according to the manufacturer’s instructions, Benelux, Netherlands) from whole blood to profile mRNA expression of 144 genes relevant for immune responses to respiratory tract infections. The genes were normalized to the housekeeping gene GAPDH, and samples with a signal below the detection level of the assay (log2-transformed peak area 7.64) were assigned to the threshold value. Genes with expression levels above the threshold in only two or fewer participants were not used for analysis. To detect differences between the patient groups, a scaled principal component analysis (PCA) plot was performed. Figures of fold change with adjusted p-value from the Wilcoxon test indicated with stars for genes with adj p-value < 0.05 and fold change >1.5 times are seen as upregulated (red) and <0.65 (1/1.5) as downregulated (blue). Comparison between severe and moderate at timepoint 1 was also analyzed with linear regression, with each gene as the response variable in the same way as CyTOF data and with covariates, age, sex, and status–timepoint. AIC was used to compare the different covariate combinations to find the best model, and the corresponding p-value from each analysis was collected and corrected with FDR. Only adjusted p-values less than 0.05 were reported.

### CXCL13 ELISA

CXCL13 levels were measured in plasma from 151 patient samples [severe T1 (n = 41), moderate T1 (n = 50), severe T2 (n = 23), moderate T2 (n = 37), and 79 healthy controls by ELISA (Human CXCL13/BLC/BCA-1 Quantikine ELISA Kit, R&D Systems Inc., Minneapolis, MN, USA)], according to the manufacturer’s instructions. The kit had a limit of detection of 3.97 pg/mL (range 7–500 pg/mL). The ELISA plates were analyzed on a Biotek EL808 plate reader at 450/562 nm using the Gen5 software. The Wilcoxon test was used to analyze the data.

### Hemagglutination inhibition

Hemagglutination inhibition (HAI) titers were measured as previously described ^74^. For season 1 samples, HAI titers against influenza A(H3N2) Switzerland, influenza A(H1N1) Cal/07/09, and influenza B Massachusetts were measured. For season 2, the same influenza A viruses were used, in addition to influenza B/Brisbane and influenza B/Phuket. For season 3, influenza A(H1N1) Michigan was added, and A(H3N2) Hong Kong replaced the Switzerland-type, while the B viruses stayed the same. For season 4, the viruses used were the same as for season 3, except for the exclusion of influenza A(H1N1) Cal/07/09. The Wilcoxon test was used to analyze the data.

### ELISPOT

Dual IFNγ/IL-2 EliSpot (CTL ImmunoSpot, Cleveland, OH, USA) was used for the detection of IFNγ- and IL-2-secreting cells as previously described (29). Frozen PBMCs were thawed, washed, and rested in tubes for 4 hours at 37°C with 5% CO_2_. A total of 200,000 cells per well were seeded and stimulated with inactivated virus A/H1N1/Cal09, A/H3N2/Shanghai, or B/Yamagata (100 HAU/mL) for 18 hours at 37°C with 5% CO_2_. A total of 400,000 cells per well were plated for peptide stimulation with CD4^+^ and CD8^+^ internal and external influenza A T-cell peptides (all 2 mg/mL). Anti-CD28 antibody was used as co-stimulation (0.1 mg/mL), and anti-CD3 antibody (0.1 mg/mL) was added as a positive control. Cytokine-positive cells were detected according to standard procedures from the manufacturer by anti-IFNγ-biotin/streptavidin and anti-IL-2-BAM/PEG [poly(ethylene glycol)-lipid biocompatible anchor for the membrane] binding and read on a CTL S6 Ultra V ImmunoSpot analyzer (ImmunoSpot, Shaker Heights, Cleveland, OH, USA). The background from unstimulated cells was subtracted for the determination of the cytokine-positive cells after stimulation. The detection limit was 0.5 units per well after background subtraction. Wilcoxon tests were performed to compare differences between the patient groups and between patients and healthy controls.

### Patient severity immune profile

An immune profile of severe disease was constructed from all outcomes significantly different between the severely and moderately ill patients at T1. To minimize the influence of outliers, the outcomes were log-transformed. All outcomes were scaled by subtracting the mean and dividing by the standard deviation. Then, a median for each participant group for all outcomes was calculated and presented in a heatmap. In addition, the scaled outcomes were correlated using Pearson’s correlation.

### Statistics

Data were analyzed using R (version 4.2.2). Comparisons between severe and moderate groups at timepoint 1 were analyzed with linear regression with gene expression as the response variable and the negative binomial regression of the number of cells in clusters from the CyTOF data with covariates, age, sex, and status–timepoint. AIC was used to compare the different covariate combinations to find the best model, and the corresponding p-value for severity differences from each analysis was collected and corrected for FDR using the Benjamini–Hochberg method. Linear regression analysis for the difference in gene expression between severe and moderate groups at timepoint 1, with the covariates age and sex, was also applied. Wilcoxon tests were performed for gene expression fold-change analysis and all other immunological analyses. For correlation analyses, Pearson’s correlation was applied. All statistical analyses reported in the text and figures are adjusted p-values, p < 0.05.

## Results

### Study participants

Ninety-one adults hospitalized due to influenza were included: 41 had severe disease and 50 had moderate disease. Seventy-nine healthy controls were included in the early autumn of 2016, prior to the influenza season. Various immunological analyses were performed using blood samples from acute infection and approximately 6 months later (at convalescence) and from healthy controls (**Figure 1**; **Table 1**). Overall, the severely ill patients were significantly older (p = 0.015) and more likely to have any underlying health conditions, such as cardiovascular, lung, kidney, liver, or neurological diseases, than the moderately ill patients (p = 0.009). Generally, there were fewer participants at follow-up from the severely ill group compared to the moderately ill group, at 56% and 74%, respectively. Only a small number of patients (n = 6) received treatment in the intensive care unit (ICU); all were part of the severely ill group. Previous CMV infection was reported to impact immune responses against influenza (30), and therefore, the incidence of prior CMV infection was analyzed, finding comparable levels between the two patient groups. Influenza type was confirmed by PCR, with A/H3N2 infection in 49 (54%) of the patients (25 severely ill and 24 moderately ill). Influenza viral load, defined as high or moderate levels, was not found to be different between the patient groups (only assessed in the CyTOF group).

**Table 1:** Demographic and clinical characteristics of study participants during acute infection at hospitalization (T1).

Characteristics	All participants					Subset of participants included in the single-cell immune profiling				
	Healthy controls	All patients	Mod¤	Sev¤¤	Mod vs. Sev	Healthy controls	All patients	Mod¤	Sev^¤¤^	Mod vs. Sev
Participants, n	79	91	50	41	NA	15	27	15	12	NA
Age, median years (range)	64 (32, 86)	69 (18, 102)	65 (18, 95)	75 (22, 102	*	66 (37, 86)	69 (20, 102)	62 (20, 83)	85 (22, 102)	***
Female, n (%)	55 (70)	49 (54)	29 (58)	20 (49)	NS	7 (47)	12 (44)	8 (53)	4 (33)	NS
Zero risk factor, n (%)	34 (43)	22 (24)	16 (32)	6 (15)	**	5 (33)	10 (37)	8 (53)	2 (17)	NS
Two or more risk factors, n (%)	16 (20)	36 (40)	15 (30)	21 (51)	NS	3 (20)	10 (37)	2 (13)	8 (67)	*
Intensive care unit (ICU) admission, n (%)	NA	6 (7)	0 (0)	6 (15)	**	NA	3 (11)	0 (0)	3 (25)	NA
Vaccinated seasonal influenza, n (%)	NA	30 (33)	17 (34)	13 (32)	NS	NA	7 (26)	4 (27)	3 (25)	NS
Influenza B, n (%)	NA	34 (37)	19 (38)	15 (37)	NA	NA	0 (0)	0 (0)	0 (0)	NA
Influenza A/H1N1^#^, n (%)	NA	6 (7)	5 (10)	1 (2)	NA	NA	0 (0)	0 (0)	0 (0)	NA
Influenza A/H3N2^#^, n (%)	NA	49 (54)	24 (48)	25 (61)	NA	NA	27 (100)	15 (100)	12 (100)	NA
Previous CMV infection, n (%)	53 (67)	66 (73)	37 (74)	29 (71)	NS	10 (67)	16 (59)	9 (60)	7 (58)	NS
High viral load, n (%)	NA	NA	NA	NA	NS	NA	20 (74)	11 (73)	9 (75)	NA

Influenza infection was confirmed by PCR.

Ctrls, controls; Mod¤, moderately ill patients; Sev^¤¤^, severely ill patients; NA, not applicable; NS, not significant; CMV, cytomegalovirus.

^#^ Two of the patients with influenza A infection were not further subtyped. They were both moderately ill.

* p < 0.05; ** p < 0.01; *** p < 0.005.

### Immune cell profiling of unstimulated cells by unsupervised analyses

**Figure 2:**
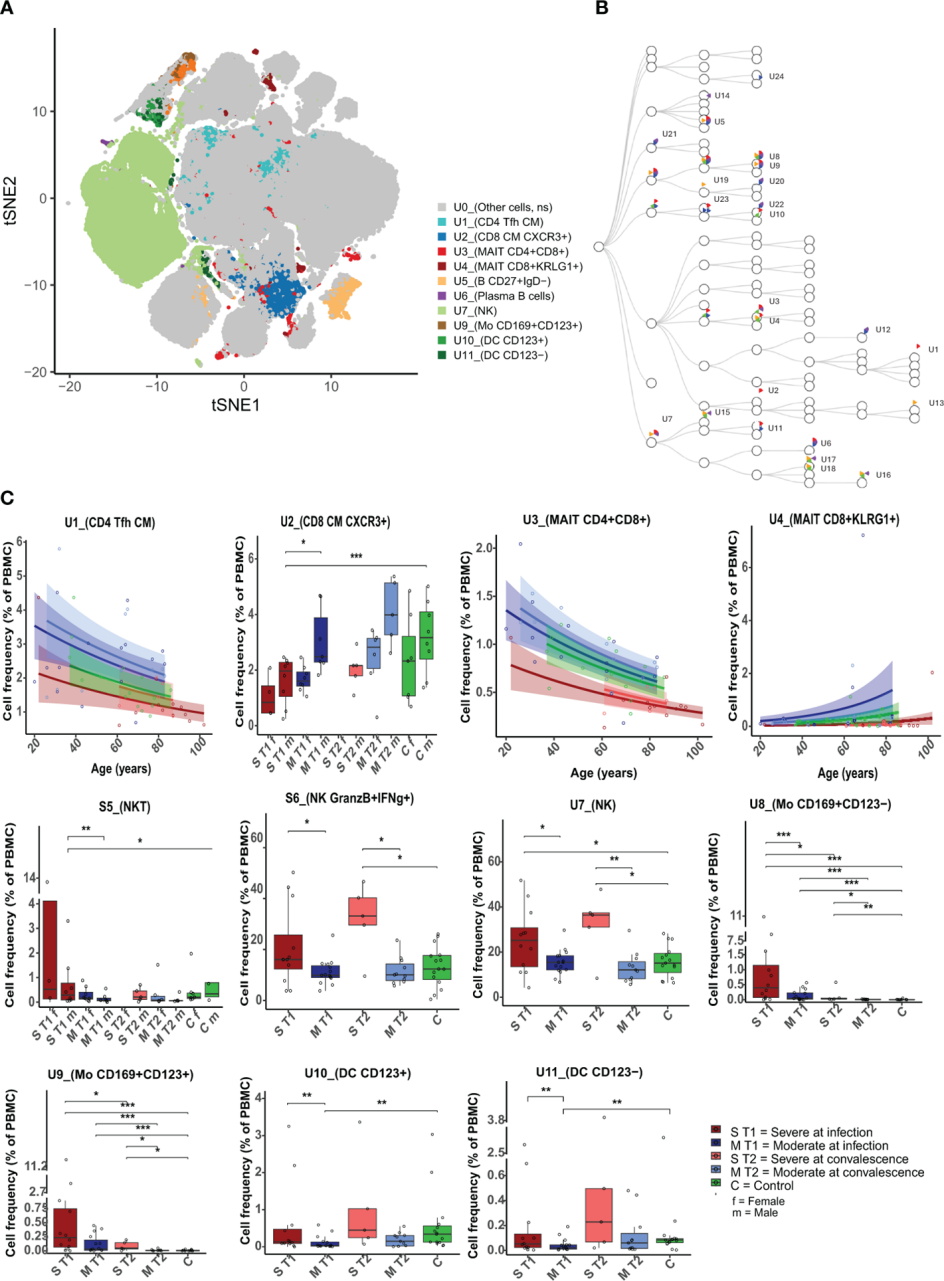
Differences in unstimulated cell population frequencies between severely and moderately ill patients. **(A)** A FlowSOM-generated t-SNE plot based on expression of all markers on cells from all samples at both timepoints (n = 59). Eleven clusters (U1–U11, illustrated by unique colors) had significantly different cell frequencies between severe and moderately ill patients during active infection (T1). **(B)** Hierarchical tree for all unique clusters (U1–U24); clusters that are significant for at least one comparison are named. Colors correspond to the group comparisons that were significant. **(C)** Boxplots/line plots of the percentage of cells per group in each of the 11 clusters from 2a. Dependency of age and sex was tested and used to define the plot type. ST1: severe during acute infection, T1 (n = 12), MT1: moderate T1 (n = 15), ST2: severe during convalescence, T2 (n = 5), MT2: moderate T2 (n = 11), C: control (n = 15), ST1F = 5 women, ST1M = 8 men, MT1F = 8 women, MT1M = 7 men, ST2M = 5 men, MT2F = 6 women, ST2M = 5 men, CF = 7 women, and CM = 8 men. There were no women among the severely ill patients at T2. Dots represent individual participants, the box indicates median with 25th and 75th percentiles, and the whiskers indicate the 1.5 × interquartile range (IQR). Line plots show the predicted value with 95% confidence interval. *adj p < 0.05, **adj p < 0.01, ***adj p < 0.001, binomial regression with FDR-adjusted for 92 clusters. Line plots show the median with predicted value and with 95% confidence interval. FlowSOM, flow self-organizing map; FDR, false discovery rate.

The characteristics of the CyTOF subgroups are presented in **Table 1**, and those at convalescence are in **Supplementary Table 1**. For the identification of cell subpopulations, data-driven unsupervised clustering was applied to samples from all participants and timepoints (**Figure 2**). The unsupervised analysis revealed 92 unique clusters (**Supplementary Figure 2A**, marker plots, **Supplementary Figure 2B**), of which 24 clusters were significantly different between all participant groups (**Figure 2A**; **Supplementary Figure 3**). When comparing patients with severe or moderate disease during acute infection, the cell frequencies were significantly different for 11 of the 24 clusters (**Figure 2B**; **Table 2**, and marker plot in **Supplementary Figure 2C**). During acute infection, the frequencies of CD4^+^ central memory (CM) T follicular helper (T_fh_) cell population [CCR7^+^CD45RA^low^ CXCR5^+^ inducible costimulatory (ICOS)^+^] (U1, i.e., unstimulated cells cluster 1), CD8^+^ CM CXCR3^+^ T cells [U2, also expressing programmed cell death-1 (PD-1) and TIGIT], MAIT cells [two subpopulations; CD4^+^CD8^+^ (U3) and CD4^−^CD8^+^ (U4, expressing KLRG1)], and a subpopulation of memory CXCR5^+^ B cells (U5) were significantly lower in the severely ill patients than in the moderately ill patients (**Figure 2C**). Further, the frequencies of CD19^low^CD38^+high^ plasma cells (U6), NK cells (U7, expressing CD16 and CD161), two subpopulations of CD14^+^CD169^+^CD85j^+^ monocytes (U8, CD123^−^ and U9, CD123^+^), and two DC populations (U10, CD123^−^ and U11, CD123^+^) were significantly higher in severe compared to moderate disease (**Figure 2C**). Older age was correlated with a reduction in the CD4^+^ T_fh_ CM T cells (U1) and MAIT CD4^+^CD8^+^cells (U3) and an increase in the percentage of the MAIT CD8^+^KLRG1^+^ cells (U4) (**Figure 2C**). Sex only affected the CD8^+^ CM T subpopulation (U2, **Figure 2C**), with men having a significantly lower frequency than women.

**Table 2:** Characteristics of unstimulated cell clusters differing at infection between severe and moderate influenza.

Cluster	Cell type	Average percentage (%) of cells across all participants	Meta cluster level	Markers characterizing the clusters and used for confirmational manual gating	Cell frequency in severe versus moderate patients	Sign. after manual gating
U*1	CD4^+^ Tfh CM	2	60	CD3^+^, CD45^+^, CD19^-^, CD4^+^, CD8^-^, CCR7^+^, CD27^+^, CD28^+^, CXCR5^+^, ICOS^+^, CD45RA^-^, TCRγδ^-^, TCRVa7.2^-^	↓	Yes
**U2**	CD8^+^ CM CXCR3^+^	2.4	30	CD3^+^, CD45^+^, CD19^-^, CD4^-^, CD8^+^, CCR7^+^, CXCR3^+^, CD27^+^, CD28^+^, CD45RA^-^, TCRγδ^-^, TCRVa7.2^-^	↓	Yes
**U3**	MAIT CD4^+^CD8^+^	0.7	30	CD3^+^, CD45^+^, CD19^-^, CCR7^+^, CD27^+^, TCRVa7.2^+^, TCRγδ^-^	↓	Yes
**U4**	MAIT CD8^+^KLRG1^+^	0.4	30	CD3^+^, CD45^+^, CD19^-^, CD8^+^, CD27^-^, CD28^-^, CD57^+^, TCRVa7.2^+^, TCRγδ^-^ , KLRG1^+^	↓	Yes
**U5**	B CD27^+^IgD ^-^	1.5	50	CD3^-^, CD45^+^, CD19^+^, CD27^+^, IgD^-^	↓	Yes
**U6**	Plasma B CD19lowCD38high	0.1	50	CD3^-^, CD45^+^, CD4^-^, CD38high, CCR4^-^, CCR6^-^, CCR7^-^, CD127^-^neg, CD134^-^, CD15^-^, CD16^-^, CD160^-^, CD169^-^, CD25^-^ , CD27^-^, CD28^-^, CD45RA^+^, CD5^-^, CD56^-^, CD57^-^, CD8^-^, CXCR3^-^, CD95^+^, HLA-DR^-^, CXCR5^-^, PD-1^-^, TCRγδ^-^, TCRVa7.2^-^, ICOS^-^, IgG^-^, IgD^-^	↑	Yes
**U7**	NK CD56^+^	18.6	10	CD3^-^, CD45^+^, CD19^-^, CD56^+^, TCRγδ^-^, TCRVa7.2^-^	↑	Yes
**U8**	Mo CD169^+^ CD123^-^	0.5	40	CD3^-^, CD45^+^, CD19^-^, CD11c^+^, CD14^+^high, CD169^+^, CD123^-^, TCRγδ^-^, TCRVa7.2^-^, CD85j	↑	Yes
**U9**	Mo CD169^+^ CD123^+^	0.3	40	CD3^-^, CD45^+^, CD19^-^, CD11c^+^high, CD14^+^high, CD169^+^, CD123^+^, CD141^+^, TCRγδ^-^, TCRVa7.2^-^, CD85j	↑	Yes
**U10**	DC CD123^+^	0.2	40	CD3^-^, CD45^+^, CD19^-^, CD11c^+^, CD123^+^, TCRγδ^-^, TCRVa7.2^-^	↑	Yes
**U11**	DC CD123^-^	0.4	30	CD3^-^, CD45^+^, CD19^-^, CD11c^+^high, CD11b^+^, CD123^-^, CD19^-^, TCRγδ^-^, TCRVa7.2^-^, CCR7^-^low, CD14^-^, CD141^+^, CD169^-^	↑	Yes

* U = significantly different cluster between moderately and severely ill patients based on **unstimulated** cells. Arrow pointing up, Higher frequency. Arrow pointing down, Lower frequency compared to moderate.

Arrow pointing up, Higher frequency.

Arrow pointing down, Lower frequency compared to moderate.

Cluster 1 (U1), consisting of CD4^+^ T_fh_ CM cells, expressed low levels of the co-inhibitory receptor PD-1, which reduces T-cell activity (30). The CD8^+^ memory T-cell cluster (U2) expressed CXCR3, indicating a memory subtype (31). Cluster 5 (U5), a CD27^+^IgD^−^ B cell cluster, indicated a memory phenotype (marker plot in Supplementary **Figure 2C**). Furthermore, the NK cell cluster (U7) expressed CD161, CD56, CD57, NKG2A, CD16, and CD38, suggesting a generally activated inhibitory NK cell population (32). Both monocyte subpopulations (U8 and U9) expressed CD169, CD141, CD14, and CD16, while U9 expressed somewhat higher CD16 in addition to CD123 (**Supplementary Figure 2C**; **Table 2**).

### Supervised gating of unstimulated cells and cell populations as potential biomarkers for severe influenza

To confirm the results from downscaled unsupervised clustering, supervised gating on all cells was performed using the most characteristic markers for the cell populations identified by the unsupervised downscaled analyses (**Table 2**). All 11 clusters were verified with significant differences in cell population frequencies according to severity during the acute infection phase (**Supplementary Figure 2D**). Further, using only one or two markers for each cell population, four of the 11 cell populations differentiating between the severely and moderately ill patients were successfully gated, suggesting that these markers may be useful as potential biomarkers for high-throughput screening of severe influenza: CD4^+^ T cells (CD4^+^; p = 0.04), MAIT (TCRVa7.2^+^; p = 0.0003), NK cells (CD56^+^CD3^−^, p = 0.02), and activated monocytes (CD169^+^CD3^−^, p = 0.0017).

We also conducted supervised gating of nine major cell populations (**Supplementary Figure 1**). Five of the nine clusters had significantly different cell frequencies in samples from patients with severe and moderate disease at T1 (CD4, MAIT, NKT, NK, and monocytes), compared to the 11 subpopulations found when using unsupervised gating.

### Immune cell profiling of stimulated cells by unsupervised analysis

**Figure 3:**
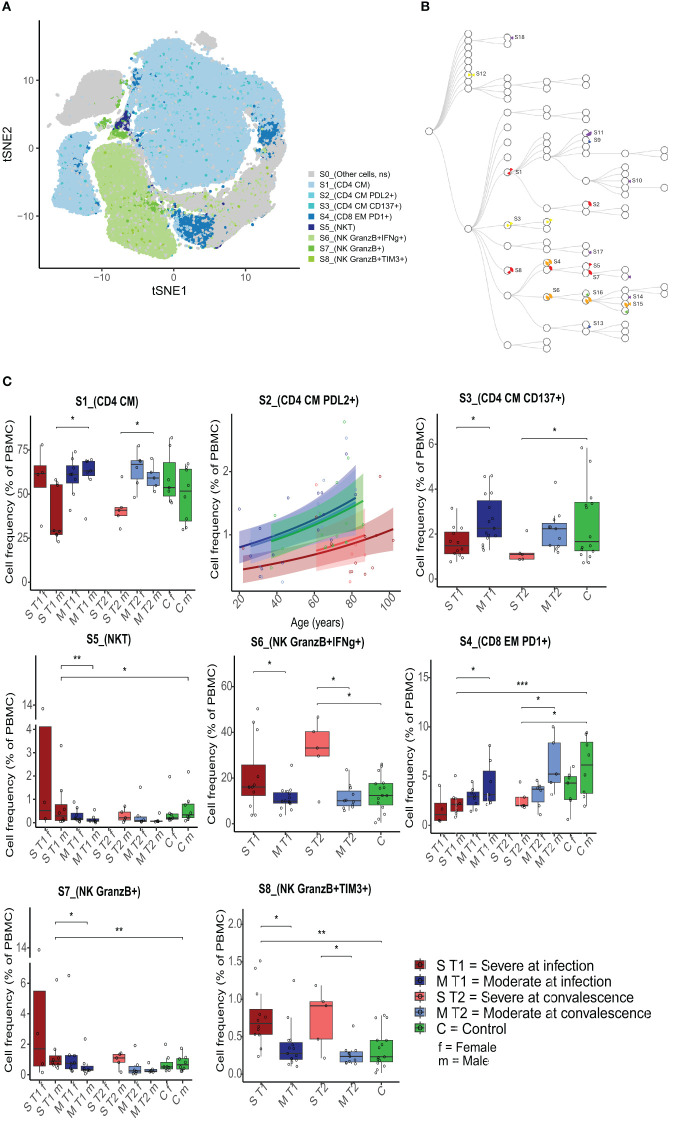
Differences in cell population frequencies after stimulation with PMA/ionomycin between severely and moderately ill patients. Eight clusters (S1 to S8) had significantly different percentages of cells per sample between severely and moderately ill patients during acute infection (T1). **(A)** t-SNE plot based on all markers and all samples at both timepoints, with the eight significant clusters illustrated by unique colors. NS = not significantly different. **(B)** Hierarchical tree for all unique clusters (S1–S18); clusters that are significant for at least one comparison are named. Colors correspond to the group comparisons that were significant. **(C)** Boxplots/line plots of the percentages of cells per group in clusters S1–S8. Dependency of age and sex was tested and used to define the plot type. ST1 = severe during acute infection, T1 (n = 12), MT1 = moderate T1 (n = 15), ST2 = severe during convalescence, T2 (n = 5) MT2 = moderate T2 (n = 11), and C = control (n = 15). There were no women among the severely ill patients at T2. Dots represent individual participants, the box indicates median with 25th and 75th percentiles, and the whiskers indicate the 1.5 × interquartile range (IQR). Horizontal lines indicate significant differences based on FDR-adjusted p-values. *adj p < 0.05, **adj p < 0.01, ***adj p < 0.001, binomial regression with FDR-adjusted for 85 clusters. Line plots show the median with predicted value and with 95% confidence interval. PMA, phorbol 12-myristate 13-acetate; FDR, false discovery rate.

Cells were stimulated with PMA and ionomycin *in vitro* to assess inherent activation capacity. The unsupervised clustering of stimulated cells from all participants and timepoints revealed 85 unique cell clusters (**Supplementary Figure 4A**). The expression of lineage markers defined the different cell types (**Supplementary Figure 3B**). In total, 18 clusters were significantly different between all participant groups (**Figure 3A**), and eight of these 18 clusters were identified with significantly different cell frequencies according to severity during acute infection (**Table 3** and **Figures 3B, C** and identification by marker plot in **Supplementary Figure 4C**).

**Table 3:** Characteristics of PMA/ionomycin-stimulated cell clusters differing between severe and moderate influenza.

Cluster	Cell type	Average percentage (%) of cells across all participants	Metacluster level	Markers defining the clusters and used for confirmatory manual gating	Sev T1 vs. Mod T1	Sign. after manual gating
S*1	CD4^+^ CM	54.3	20	CD3^+^, CD45^+^, CD19^−^, CCR7^+^, CD25^+^, CD27^+^, CD45RA^−^, TCRγδ^−^	↓	Yes
S2	CD4^+^ CM PD-L2^+^	1.1	40	CD3^+^, CD45^+^, CD19^−^, CCR7^+^, CD25^+^, CD27^+^, CD28^+^, CD45RA^−^, TCRγδ^−^, PD-L2^+^	↓	Yes
S3	CD4^+^ CM CD137^+^	2.2	20	CD3^+^, CD45^+^, CD19^−^, CD27^+^, TCRγδ^−^, CD137^+^	↓	Yes
S4	CD8^+^ EM PD-1^+^	3.6	30	CD3^+^, CD45^+^, CD19^−^, CD27^+^, CD28^+^, CD45RA^−^, TCRγδ^−^, TNFα^+^, IL-1β^+^, MIP-1β^+^, IFNγ^+^, CD107a^+^	↓	Yes
S5	NKT CD3^+^CD56^+^	0.6	50	CD3^+^, CD45^+^, CD19^−^, CD57^+^, CD28^−^, CD45RA^+^, CCR7^−^, TCRγδ^−^, TNFα^−^, PD-1^+^, GranzB^+^, CD107a^+^	↑	Yes
S6	NK CD56^+^ GranzB^+^ IFNγ^+^	15.6	30	CD3^−^, CD45^+^, CD19^+^, CD56^+^, GranzB^+^, CD107a^+^, IFNγ^+^, CD57^+^, MIP-1β^+^, TNFα^+^, TCRgd^−^	↑	Yes
S7	NK CD56^+^ GranzB^+^	1.2	50	CD3^−^, CD45^+^, CD19^−^, CD56^+^, GranzB^+^, CD107a^+^, IFNγ-, TCRγδ^−^, CD38^+^	↑	Yes
S8	NK CD56^+^ GranzB^+^ TIM3^+^	0.5	20	CD3^−^, CD45^+^, CD19^−^, CD56^+^, GranzB^+^, CD107a^+^, TNFα^+^, MIP-1β^+^, TIM3^+^, CD57^+^, TCRγδ^−^	↑	Yes

* S, significantly different clusters between moderately and severely ill patients based on stimulated cells.

PMA, phorbol 12-myristate 13-acetate.

Arrow pointing up, Higher frequency. Arrow pointing down, Lower frequency compared to moderate.

We observed a lower frequency of several CD4^+^ CM cell subsets [stimulated cells, clusters 1 (S1), S2 (included in S1), and S3] and a CD8^+^ effector memory T-cell subset (TEM) (S4) in the severely ill patients compared to the moderately ill patients at infection (**Figure 3B**). Moreover, in the severely ill patients, a higher frequency of cells was found in a cluster containing both NKT and CD8^+^ terminally differentiated effector memory T cells (TEMRA) (S5) and in three NK cell clusters (S6, S7, and S8) at infection (**Figure 3B**). S1, the largest CD4^+^ CM T-cell cluster (average 54.5% in all participants), contained S2 but not S3 and was negative for CD137/4IBB. S2 expressed CD272/PD-L2 (programmed cell death 1 ligand 2), an immune checkpoint for the negative regulation of the adaptive immune response (33), and S3 expressed CD137/4-IBB [tumor necrosis factor receptor (TNFR) superfamily member], suggesting antigen-activated T cells (34) (**Supplementary Figure 3C**). The NKT/TEMRA subpopulation (S5) expressed high levels of granzyme B (GranzB) and CD107a, and the CD8^+^ EM T cells (S4) expressed high levels of several cytokines (TNFα, IFNγ, and IL-10) and the chemokine MIP-1β, while GranzB expression was low. Among the three NK cell subsets, S6 included most of all NK cells (average of 15.6% in all participants) and together with S8 contained high levels of CD57^+^ cytokine-secreting cells (GranzB, IFNγ, TNFα, IL12p-70, perforin, IL-1β, IL-2, and chemokine MIP-1β). In addition, NK cell cluster S8 expressed TIM-3 (T-cell immunoglobulin and mucin domain 3), a negative regulator of Th1-type immune responses. The third NK cluster (S7) expressed lower levels of CD57 and fewer cytokines, but higher CD38 expression (mediating NK cell activation (35)) compared to S6 and S8. Women had a higher frequency of cells in cluster S7 than men (**Figure 3B**), and they had higher frequencies of CD4^+^ CM T cells (S1) and GranzB-secreting NKT cells (S5) and a lower frequency of CD8^+^ EM PD-1^+^ and GranzB^low^ T cells (S4) than men. There was a significant increase in the frequency of CD4^+^ CM PD-L2^+^ T cells (S2) with increasing age (**Figure 3C**). We confirmed the significant difference in cell population frequency between the patient groups in all eight clusters by supervised gating using the most prominent markers (**Table 3**; **Supplementary Figure 3D**).

### Immune cell population differences between all participant groups

**Figure 4:**
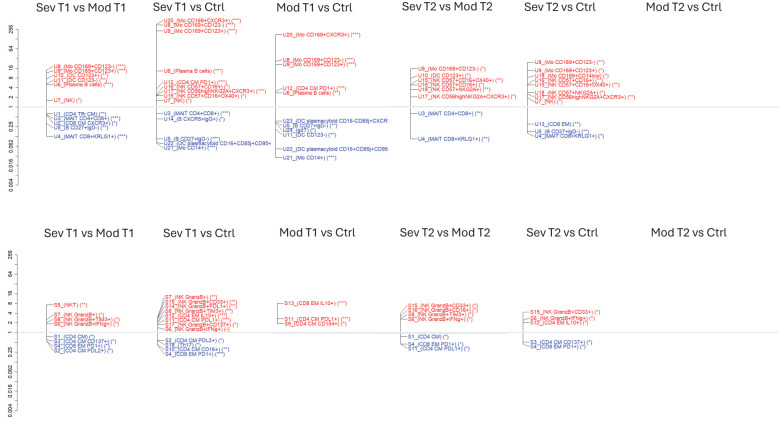
Differences in cell population frequencies between all participant groups during acute infection and convalescence. Incident rate ratio (IRR) for all clusters with significantly different cell population frequencies between severe, moderate, and control groups during acute infection (T1) or convalescence (T2) in unstimulated **(A**–**F)** and stimulated **(G**–**L)** cells based on negative binomial regression analysis. Red represents cell subpopulations with higher IRR, and blue represents cell subpopulations with lower IRR. FDR-adjusted p-values: *adj p < 0.05, **adj p < 0.01, *** adj p < 0.001. ns = no significant difference between healthy controls and moderate at convalescence (f and l). FDR, false discovery rate.

We identified the cell cluster differences between all participant groups (severely and moderately ill patients and controls) and different timepoints (acute and convalescent phases) and reported the IRRs for significant comparisons (**Figure 4**). We identified the significant clusters using marker plots (**Figures 2B**, **3B**; **Supplementary Figure 4**). The comparison of severe and moderate disease at T1 is also shown in **Figures 2** and **3**.

Comparing patient groups at the time of acute infection to healthy controls, there were 14 clusters with different frequencies between the severely ill patients and controls (**Figure 4B**) and 11 between the moderately ill patients and controls during acute infection (**Figure 4C**). Subpopulations of monocytes, plasma cells, and CD4^+^ CM/T_fh_ PD-1^+^ T cells appeared more frequently in both patient groups, while memory B cells, CD14 monocytes, and plasmacytoid DC were less frequent in both patient groups (**Figures 4B, C**).

In total, there were eight significantly different clusters of cell populations in the severely compared to moderately ill patients during convalescence (**Figure 4D**). The severely ill patients still had lower frequencies of MAIT subpopulations and higher frequencies of NK, monocyte, and dendritic subpopulations. Also at convalescence, the severely ill patients had different frequencies of 11 cell subpopulations compared to controls (**Figure 4E**). A higher frequency of CD169^+^ monocyte and CD161^+^ NK cell subpopulations and a lower frequency of MAIT subpopulations and memory B cells were identified in the severely ill patients compared to controls. In contrast, the moderately ill patients had no different subpopulations compared to the controls.

Comparing the cluster frequencies of the stimulated cells during acute infection, there were more subpopulations with different frequencies between the severely ill patients and the controls (12) (**Figure 4G**) than between the moderately ill patients and the controls (3) (**Figure 4H**). The CD4^+^ T-cell signaling differed upon severity, with a higher frequency of a cluster containing polyfunctional (IFNγ and TNFα) PD-1^+^ IL-10-secreting CD4^+^ effector memory T cells and regulatory T cells in the severely ill patients than in the controls (**Figure 4G**). A higher frequency of activated CD154^+^ and suppressive PD-L1^+^ CD4^+^ T cells was observed in the moderately ill patients than in the controls (**Figure 4H**). The frequency of Th17 cells was lower in the severely ill patients compared to the healthy controls, but this was not the case for the moderately ill patients (**Figures 4G, H**, respectively).

The comparison of cell frequencies at convalescence revealed lower frequencies of two CD4^+^ CM subpopulations and one CD8^+^ EM subpopulation and higher frequencies of four NK subpopulations in the severely compared to moderately ill patients (**Figure 4J**). Further, the frequencies of subpopulations of CD4^+^IL-10^+^ EM T cells and NK cells (CD33^+^GranzB^+^ and GranzB^+^/IFNγ^+^) remained higher in the severely ill patients than in the controls, while the frequencies of CD4^+^ CM T and CD8^+^ EM T subpopulations were lower (**Figure 4K**). The latter subpopulations were the same as those identified to be lower in stimulated cells from the severely compared to moderately ill patients during acute infection (**Figure 4G**), while Th17 cell frequency no longer differed between the severely ill patients and controls during convalescence (**Figure 4K**). Furthermore, the cell frequencies in moderate disease normalized to levels in healthy controls during convalescence, as no difference in cell subpopulations was found in both unstimulated and stimulated cells (**Figures 4F, L**).

### Differential gene expression analysis

**Figure 5:**
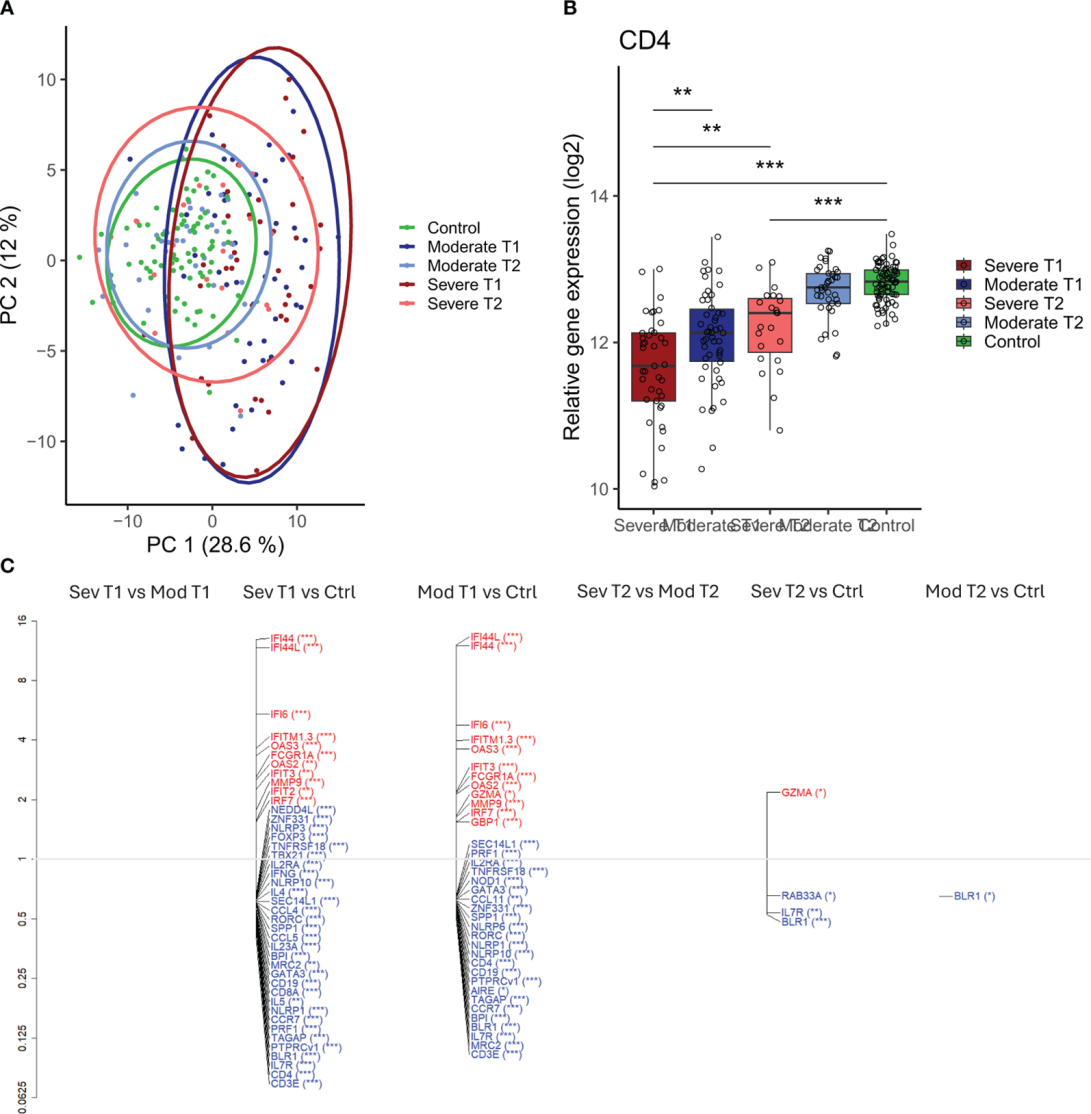
Differential gene expression in moderately and severely ill patients compared to healthy controls. **(A)** Scaled principal component analysis (PCA) plot representing individuals, status, and timepoint. **(B)** *CD4* gene expression in severely and moderately ill patients at infection and convalescence and in healthy controls. Each point represents an individual, box plot shows group median with 25th and 75th percentiles, and whiskers indicate 1.5 × interquartile range (IQR). Differences were analyzed by linear regression analysis. **(C)** Differentially expressed genes (DEGs) at infection and convalescence between all participant groups. The vertical axis (y-axis) corresponds to the fold change (axis on log2 scale), and the FDR-adjusted p-values are indicated as significance levels in parentheses. Red color represents genes with higher expression, and blue color represents genes with lower expression. N = 230 (severe at infection, n = 41; moderate at infection, n = 50; severe at convalescence, n = 23; moderate at convalescence, n = 37; control, n = 79). *adj p < 0.05, **adj p < 0.01, ***adj p < 0.001. IQR, interquartile range; FDR, false discovery rate.

We further conducted a dcRT-MLPA assay for transcriptomic analysis to explore potential variations at the gene expression level between the severely (n = 41) and moderately (n = 50) ill influenza patients, compared to the healthy controls (n = 79) (**Table 1**). A scaled PCA plot of 128 genes (**Supplementary Table 6**) showed that the gene expression profile in the severely and moderately ill patients during acute infection was similar and partially separated from the gene expression of the healthy controls and from both patient groups during convalescence (**Figure 5A**). At convalescence, the gene expression of the patient groups resembled that of the controls (**Figure 5A**).

When comparing severe and moderate influenza patients, there were no differentially expressed genes during either acute infection or convalescence based on a combination of the Wilcoxon test and fold-change analysis (**Figure 5C**). However, applying linear regression analysis with adjustment for age and sex revealed that *CD4* expression was significantly lower in the severely compared to moderately ill patients during acute infection only (adj p = 0.018, **Figure 5B**).

Forty-two and thirty-six genes were found to be differently expressed during acute infection between controls and the severely or moderately ill patients, respectively (adj p < 0.01–0.001) (**Figure 5C**). Thirty of these genes were shared between the severe and moderate disease groups (data not shown). Most of the upregulated genes during acute infection were IFN-signaling genes (*IFI44*, *IFI6*, and *IFITM1.3*), while most of the downregulated genes belonged to the adaptive immune response corresponding to typical T-cell and B-cell subsets (*CD3E*, *CD4*, and *IL-7R*) (**Supplementary Figure 5C**). The gene expression patterns in the convalescent phase were similar to expression seen in the healthy controls for both patient groups (**Supplementary Figure 5C**); however, four (*BRL1*, *IL7R*, *RAB33A*, and *GZMA*) and one (*BRL1*) differentially expressed genes were found in the severely and moderately ill patients, respectively, compared to healthy controls.

Acute gene expression in patients infected with influenza A/H3N2 (n = 49) and influenza B (n = 34) was not significantly different, thus not affected by the infecting influenza strain (data not shown).

### Influenza-specific humoral and cellular responses

There were no differences in acute or convalescent HAI titers due to the severity of any of the viruses (data not shown). HAI titers were induced after infection, and titers were the highest at convalescence in A/H3N2-infected patients (p = 0.001, n = 28 paired observations, 13 severe and 15 moderate; Wilcoxon) but not after influenza A/H1N1 (n = 23 paired observations, eight severe and 15 moderate) or B infection (n = 5 paired observations, one severe and four moderate).

We also analyzed influenza-specific cellular responses by stimulating PBMCs with either homologous inactivated whole virus [A/H3N2, A/H1N1(Cal09), or B virus] or peptide panels representing conserved influenza CD4^+^ and CD8^+^ T-cell epitopes in the dual IFNγ and IL-2 EliSpot assay. No significant differences between the severely and moderately ill patients were found at any timepoint (data not shown).

### Plasma CXCL13 levels

CXCL13 levels in plasma were measured as a biomarker for activated B and T cells in germinal centers contributing to antibody production (27). Acute CXCL13 levels were significantly higher in the severely ill patients (n = 41) compared to the moderately ill patients (n = 50) (p < 0.05, Wilcoxon test) and also significantly higher than during convalescence, which were similar in both patient groups and controls (n = 79) (**Supplementary Figure 5**). The CXCL13 values were not affected by sex or age at either timepoint.

### Integrated analysis to define an immune profile associated with severe influenza

By integrating all the significant findings between the severely and moderately ill patients at acute infection into a heatmap (n = 21), we defined a severity immune profile (**Figure 5A**). The results during convalescence were also included in the heatmap. The heatmap shows a distinct immune profile for the severely ill patients at acute infection compared to the moderately ill patients and healthy controls. During convalescence, the severely ill patients had higher similarities to their profile at acute infection, whereas the convalescent profile of the moderately ill patients more closely resembled that of the healthy controls. This indicates that some of the cell population alterations during acute infection in the severely ill patients were still present at convalescence.

**Figure 6:**
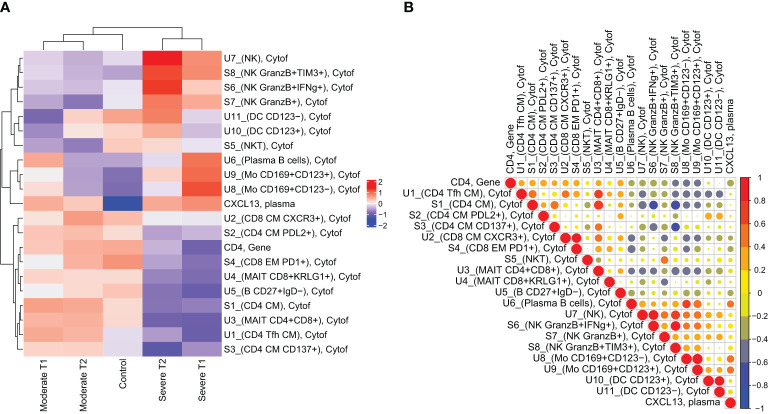
Immune profile of influenza disease status and correlation analysis of results in severely ill patients. **(A)** Severity immune profile based on the median value per outcome significantly different between moderate and severe patients during acute infection (T1) (21 different outcomes). These outcomes are presented for all participant groups during acute infection (T1) and convalescence (T2) (severe T1, n = 10; moderate T1, n = 12; severe T2, n = 4; moderate T2, n = 10; controls, n = 15). Percentages of immune cell subsets and plasma CXCL13 levels are log-transformed, differential gene expression is log2-transformed and scaled, and a participant group median is shown in the heatmap. **(B)** Correlation matrix of the 21 significant outcomes at T1, presented for the severely ill patients (ST1 = severe T1, n = 10).

To investigate the relationship between the 21 putative biomarkers, we performed a correlation analysis of all variables significantly different at acute infection between the severely and moderately ill patients for both patient groups and controls (**Figure 6** for severe cases and **Supplementary Figure 6** for the moderately ill patients and controls). A strong positive correlation was demonstrated both between circulating plasma B cells and CXCL13 levels in plasma and between CD4^+^ T cells and MAIT cells in the severe patients (**Figure 5B**). In these patients, *CD4* gene expression correlated with CD4^+^ and CD8^+^ population frequencies, and there was a negative correlation between *CD4* expression and monocyte subpopulation frequencies, DCs, and NK subpopulations (U8 and U9 unstimulated monocytes, U10 and U11 unstimulated DCs, and S6 stimulated cytokine-producing NK cells). Moreover, we found that NK cell frequencies (U7 unstimulated cells and S6 and S8 stimulated cells) were positively correlated with monocytes (U8 and U9 unstimulated cells) and DCs (U10 and U11 unstimulated cells) in the severe group.

## Discussion

Influenza poses a global health challenge, and identifying immune biomarkers for disease severity may guide clinical follow-up and future vaccination strategies. In this study, we identified distinct immune cell profiles differing between severe and moderate influenza disease. All patients included were hospitalized, albeit mostly not critically, with only a few requiring ICU treatment. Analyses of bulk gene expression revealed only *CD4* as differentially expressed upon severity, while unsupervised single-cell mass cytometry analysis identified several subpopulations of immune cells with different frequencies according to severity; therefore, single-cell assessment offers a more powerful approach to characterize and compare disease states. Despite a large inter-individual variation of immune cell populations usually found in healthy individuals (36), we identified immune cell subpopulations differentiating between moderate and severe influenza in hospitalized individuals.

Overall, our findings suggest that disease severity is associated with alterations in the immune response, with a reduction in memory-like T-cell responses and an increase in proinflammatory phenotypes, including inhibitory monocytes and NK cells. During convalescence, the severely ill patients maintained proinflammatory immune cell composition, while the cell subpopulations of patients with moderate disease resembled those of healthy controls. This insight contributes to a deeper understanding of the immune cell dynamics during influenza infection and long-term effects.

The association between lower levels of memory cell responses and disease severity supports prior research demonstrating the protective influence of certain immune cell subsets against symptomatic influenza. Beneficial effects have been shown for CXCR5^+^CD4^+^ T_fh_ cells (37, 38), CD137^+^CD4^+^ and CXCR3^+^CD8^+^ CM T cells (39), and CD27^+^IgD^−^ memory B cells (40, 41). In accordance with this, we observed a functional difference in CD4^+^ subpopulations. Specifically, patients with moderate illness had higher frequencies of activated CD137^+^CD4^+^ CM T cells, where CD137 expression previously has been associated with antigen-specific responses (42). Additionally, we found an increased frequency of PD-L2^+^CD4^+^ CM in the same patient group. Engagement of PD-L2 has been reported to mediate negative feedback and the downregulation of excessive cytokine production (33), possibly indicating a beneficial response in the moderately ill patients compared to the severely ill patients.

The lower frequencies of MAIT cells in patients with severe disease aligned with previous findings, linking the fatal outcome of influenza disease to decreased numbers of MAIT cells (23). Consistent with this, studies investigating severe influenza and COVID-19 have confirmed reduced counts of circulating MAIT as well as CD4^+^ and CD8^+^ T cells (43, 44). This may reflect the migration of immune cells to the infection site in the lungs, as evidenced by elevated numbers of T and B cells detected in bronchoalveolar lavage (BAL) samples from both influenza and COVID-19 patients (12, 23, 45). Since KLRG1 signaling can inhibit the proliferation and function of immune cells, a reduction in KLRG1^+^ MAIT cells in severe influenza may reflect a dysregulation of immune cell proliferation (46). A similar proportion of CMV infection in the patient groups does not suggest any association between severity and CMV infection, and a previous study even showed that CMV infection was associated with increased influenza A-specific T-cell responses early upon acute influenza infection (47). Seasonal influenza vaccination rate was low (approximately 25%) and similar in both patient groups and therefore does not seem to be a reason for the different memory T and B subpopulation distribution, even though there are most likely large individual variations in vaccine responses, as previously shown in T-cell subpopulation distribution (48).

The diminished expression of the *CD4* gene in severe cases during infection is in line with previous studies (49) and supports our observation of reduced numbers of circulating CD4^+^ cells. While *CD4* was the only gene differing upon severity in our study, both patient groups exhibited upregulation of IFN-signaling genes indicative of the innate immunity activation and downregulation of genes linked to adaptive immune responses during acute infection, consistent with earlier findings (16, 49). The absence of differences in the expression of genes related to neutrophil activation, antimicrobial responses, and inflammatory pathways between the patient groups in our study may reflect heterogeneity in severity definition, severity grade, and possible variations in patient sampling timepoints across studies. Despite a comparable gene expression pattern between the two patient groups, mass cytometry identified 11 cell subpopulations distinguishing between severe and moderate disease, again illustrating the sensitivity of high-dimensional single-cell analyses as opposed to bulk gene expression.

NK and NKT cells play a crucial role in connecting innate and adaptive immunity, triggering antigen-specific T- and B-cell responses, aiding viral clearance, and acting as primary cytokine producers. This cytokine production, when excessive, may contribute to immunopathology, as seen during a cytokine storm (50–52). Our study revealed elevated frequencies of cytokine-producing NK and NKT cells in severely ill patients, including the main population of GrzB^+^IFNγ^+^ NK cells and the subpopulations of GrzB^+^IFNγ^−^ and GrzB^+^IFNγ^+^TNFa^+^TIM-3^+^NK cells. The upregulation of TIM-3 on NK/NTK cells suggests a negative feedback mechanism, leading to the inhibition of cytokine production in the target cytotoxic T cell (52). This may reduce the excessive NK cell activation and the risk of cytokine storm and alleviate immunopathology, as shown in a mouse model where enhanced TIM-3 activity improved survival after influenza infection (53). Notably, previously reported frequencies of NK and NKT subpopulations in severe influenza cases show inconsistency (12, 13, 51).

In our study, severity was also found to be associated with elevated frequencies of monocytes (Mo) CD169^+^CD14^+^CD16^+/low^CD141^+^CD85j^+^CD123^+/−^, as well as CD123^+/−^ DC subpopulations. Monocytes bearing these markers are recognized as activated and proinflammatory (54–56). In addition, cross-linking of CD85j inhibits the activation of B cells, T cells, NK cells, and macrophages (57). Increased frequency of intermediate monocytes has been reported after influenza infection (25), suggesting a potential inflammatory response (58, 59). Older age has also been associated with a higher frequency of classical CD14^+^CD16^−^ monocytes, and specifically, an increased expression of activation markers on monocytes and dendritic cells was associated with a frailty phenotype (60). Consistent with our results, the severity of COVID-19 has also been linked to proinflammatory monocyte populations expressing CD169, which is upregulated upon activation and serves as an early marker for SARS-CoV-2 infection in patients requiring oxygen treatment (54, 61–63). CD141 expression was previously shown to be increased in monocytes in both moderate and severe COVID-19 cases (64), while CD123 was upregulated in severely ill COVID-19 patients requiring ventilation (65). The lower frequencies of the identified DC populations at MT1, but not MT2 (in the moderately ill group compared to the control group), may reflect a redistribution of immune cells to the lungs during active infection. This is in accordance with the reduced levels of several other cell subpopulations at T1 compared to T2 (memory B cells, plasmacytoid DC, and classical monocytes) and supported by previous reports (66). A similar shift in the severely ill group seemed to be present as well; however, higher frequencies of these DC populations were seen in the severely ill patients compared to the moderately ill patients at both T1 and T2. Comorbidities may account for the increase in plasmacytoid DC frequency (67, 68), although they are often linked to a reduced DC frequency (60, 69).

Taken together, the increased frequencies of specific subpopulations of NK, NKT, Mo, and DC observed in severe influenza infection may suggest a more proinflammatory state, possibly exacerbated by advanced age and underlying risk factors. Compared to healthy controls, we observed higher frequencies of circulating plasma cells in both patient groups during infection, with the highest frequency observed in the severe cases. This is in line with studies of COVID-19 disease, where patients with peripheral plasma cells had a higher likelihood of severe clinical outcomes (70). In the severely ill patients, plasma cell frequencies correlated with plasma CXCL13 levels. Elevated plasma CXCL13 levels have previously been linked to antibody production after influenza vaccination (27). For influenza A/H3N2 patients, HAI titers were significantly higher during convalescence, indicating active antibody production. However, no differences in specific antibody titers were observed due to severity, which is in line with a previous influenza study (71) but contradictory to severe COVID-19 disease (72, 73).

The severely ill patients in our study were older and had more risk factors compared to the moderately ill patients. High frequencies of Mo, NK, and NKT cells and especially low MAIT cell frequencies during influenza have been linked to frailty and elevated risk of severe disease, supporting the findings in our study (12). An increase in the frequency of a CD14^−^CD16^high^ monocyte population was present in post-COVID-19 conditions (74), whereas we identified an increase in CD14^+^CD16^+/low^ monocytes. Somewhat conflicting data exist on monocyte subpopulation distribution and age since an increase in the frequency of both CD14^+^CD16^−^ and CD16^+^ Mo subpopulations as well as NK cells was reported to be associated with age, while the plasmacytoid DC population is reduced in both size and capacity with higher age (55, 60, 75, 76). The immune responses observed in the elderly have been associated with a decline in the frequency of lung-resident memory T cells and circulating memory B-cell frequency (12, 39). We also identified a general reduction in the CD4^+^CD8^+^ MAIT subpopulation with advancing age, in agreement with previous research (77, 78). Conversely, the CD4^−^CD8^+^ MAIT subpopulation increased with age. Nevertheless, age alone could not explain the observed differences in immune cell profiles in relation to severity.

Certain features of severe disease, such as lower frequencies of MAIT and memory T and B cells, as well as elevated frequencies of proinflammatory NK cells and monocytes, persisted into the convalescence phase. At this timepoint, 6 months after active infection, we expected that the immune status had returned to baseline as observed in the moderately ill patients, but this was not the case for the severely ill patient group. However, differences in cell population frequencies may have existed prior to infection, indicating that a pre-infection immune profile could also serve as a predictor of influenza severity (79). The lower frequency of CD8 EM cells in severely ill patients compared to healthy controls at convalescence may be due to a higher number of comorbidities in the influenza patients, indicating frailty (80). Alternatively, our findings may reflect that severely ill patients need a longer time to return to a normal state after a prolonged activation phase (71), as seen after SARS-CoV-2 infection (81). A reduction in CD4^+^ CM cells and an increase in non-conventional monocytes (CD14^low^CD16^high^) was, however, also present in post-COVID-19 conditions (74). Increased proinflammatory phenotypes could also indicate low-grade chronic infection, as previously reported, especially in older individuals with multiple risk factors (82). Persisting low-grade infection is characterized by higher frequencies of IL-10-secreting CD4^+^ effector memory T cells (83), which agrees with our findings in the severely ill patients in both the acute and convalescent phases. Elevated *granzyme A* expression and frequency of NK cells in severely ill patients at convalescence may reflect an ongoing infection or delayed recovery, as reported previously (84, 85).

The high number of phenotypically and functionally distinct cell populations distinguishing severe and moderate disease highlights the superior sensitivity of high-dimensional immune cell profiling at the single-cell level, as opposed to bulk information from gene expression analysis. We were able to identify phenotypically and functionally distinct cell populations as candidate biomarkers for severe influenza. Manual gating of these cell populations using a considerably smaller number of markers verified the populations as different in severe disease. Additionally, when gating CD4^+^ T cells, MAIT cells, NK cells, and activated monocytes using only one to two markers for each population, this difference remained significant, which strengthens our observations and suggests that these could be further explored as potential biomarkers for severe influenza in a clinical setting. This would also allow the use of less comprehensive screening methods, like high-throughput flow cytometry.

A limitation of this study is the relatively low number of participants and particularly few patients in the ICU subgroup, reducing the difference between the patient groups, which probably limited the predictive power of our immune profile. However, strong correlations between *CD4* gene expression, CXCL13 plasma levels, and specific immune cell clusters supported our findings from mass cytometry analysis. Due to the small sample size, we were unable to test the interaction of sex or age with disease status–timepoint. However, when the implication of sex or age is additive, i.e., was similar for all the disease status–timepoint, this was included in the analysis. The analyses were only presented when differences between severity groups were identified. Longitudinal analyses, i.e., analyses within individuals at T1 and T2, were not included due to the few samples at T2 in the severely ill group. A higher number of samples would increase the robustness of the findings and possibilities for specific age- and sex-associated analyses.

Moreover, the immune cell population differences identified in this study are based on H3N2 infection but may also be relevant for other influenza strains due to epitope conservation (e.g., 57% of CD4 and 63% of CD8 epitopes are either identical or conserved when comparing seasonal IAV and H5N1 (86)).

In conclusion, this study illustrates the power and high sensitivity of high-dimensional single-cell analyses compared to bulk analyses in the identification of functional cell population frequencies as potential biomarkers for disease severity. The immune profile characteristic of severe influenza included lower frequencies of MAIT cells and memory B- and T-cell subpopulations, with higher frequencies of proinflammatory NK cells, monocytes, dendritic cells, and plasma cells. Interestingly, the severity-associated alterations persisted at convalescence, possibly accelerated by higher age and the presence of several underlying conditions, inducing frailty. Some immune signatures of post-COVID-19 conditions overlap with the long-term immune features we present here of severe influenza. Although the clinical usefulness of the severity profile identified here requires further evaluation in future larger studies, reduced CD8 immunity associated with severe influenza disease suggests that vaccination strategies like mRNA vaccines directed to improve CD8 responses may be advantageous to improve protection against severe influenza.

## Data Availability

The raw data supporting the conclusions of this article will be made available by the authors, without undue reservation.
