# Correction: High-dimensional single-cell phenotyping unveils persistent differences in immune cell profiles between severe and moderate seasonal influenza

**DOI:** 10.3389/fimmu.2025.1685215

**Published:** 2025-08-19

**Authors:** Frontiers Production Office

**Affiliations:** Frontiers Media SA, Lausanne, Switzerland

**Keywords:** influenza, hospitalization, disease severity, mass cytometry, immune profiling, biomarkers

The figures were in the wrong order. **Figure 2** and **Figure 5** were displayed incorrectly and should be swapped. The correct **Figure 2** and **Figure 5** appear below. The order has now been corrected.

**Figure 2:**
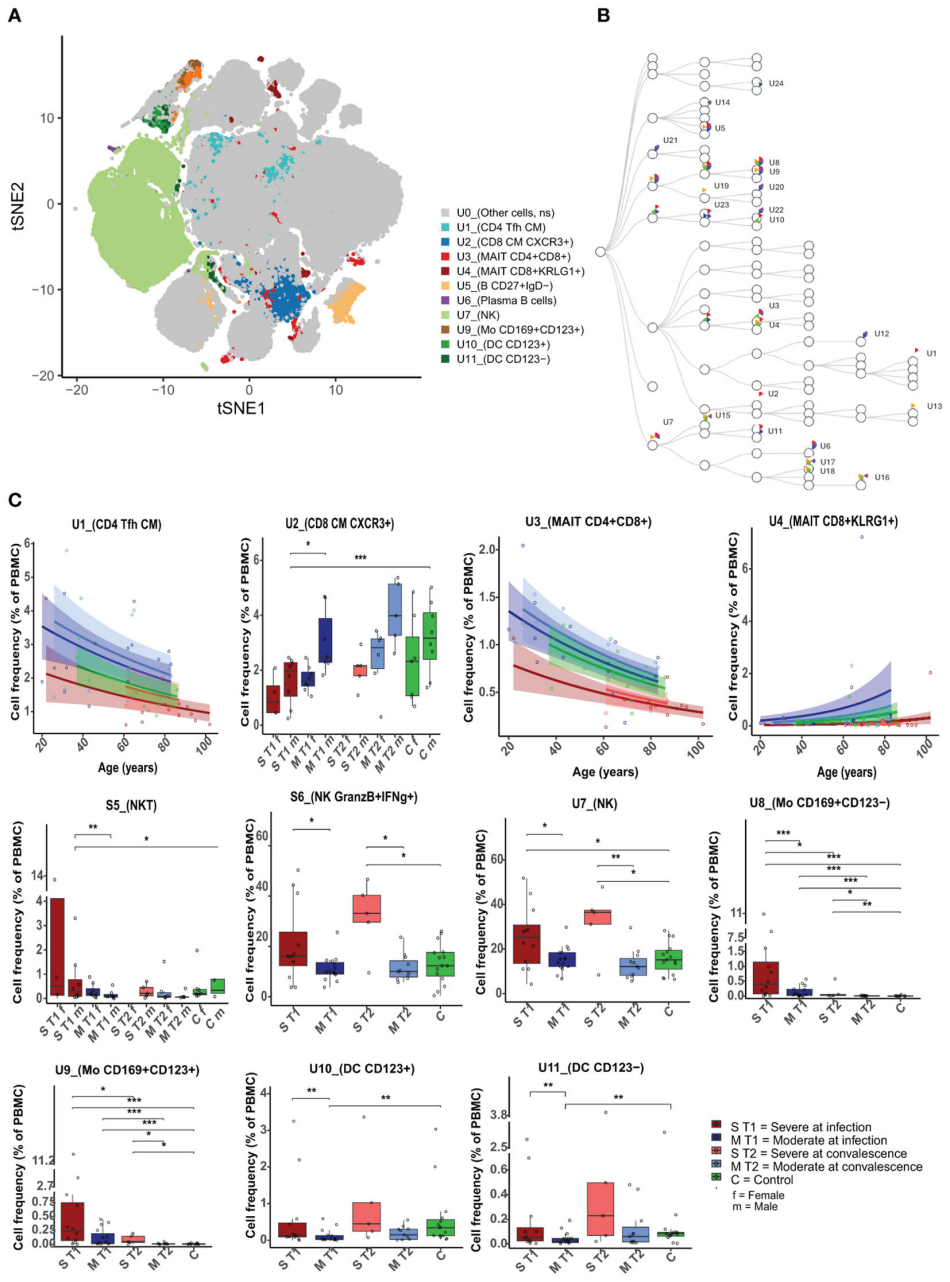
Differences in unstimulated cell population frequencies between severely and moderately ill patients. **(A)** A FlowSOM-generated t-SNE plot based on expression of all markers on cells from all samples at both timepoints (n = 59). Eleven clusters (U1–U11, illustrated by unique colors) had significantly different cell frequencies between severe and moderately ill patients during active infection (T1). **(B)** Hierarchical tree for all unique clusters (U1– U24); clusters that are significant for at least one comparison are named. Colors correspond to the group comparisons that were significant. **(C)** Boxplots/line plots of the percentage of cells per group in each of the 11 clusters from 2a. Dependency of age and sex was tested and used to define the plot type. ST1: severe during acute infection, T1 (n = 12), MT1: moderate T1 (n = 15), ST2: severe during convalescence, T2 (n = 5), MT2: moderate T2 (n = 11), C: control (n = 15), ST1F = 5 women, ST1M = 8 men, MT1F = 8 women, MT1M = 7 men, ST2M = 5 men, MT2F = 6 women, ST2M = 5 men, CF = 7 women, and CM = 8 men. There were no women among the severely ill patients at T2. Dots represent individual participants, the box indicates median with 25th and 75th percentiles, and the whiskers indicate the 1.5 × interquartile range (IQR). Line plots show the predicted value with 95% confidence interval. *adj p < 0.05, **adj p < 0.01, ***adj p < 0.001, binomial regression with FDR-adjusted for 92 clusters. Line plots show the median with predicted value and with 95% confidence interval. FlowSOM, flow self-organizing map; FDR, false discovery rate.

**Figure 5:**
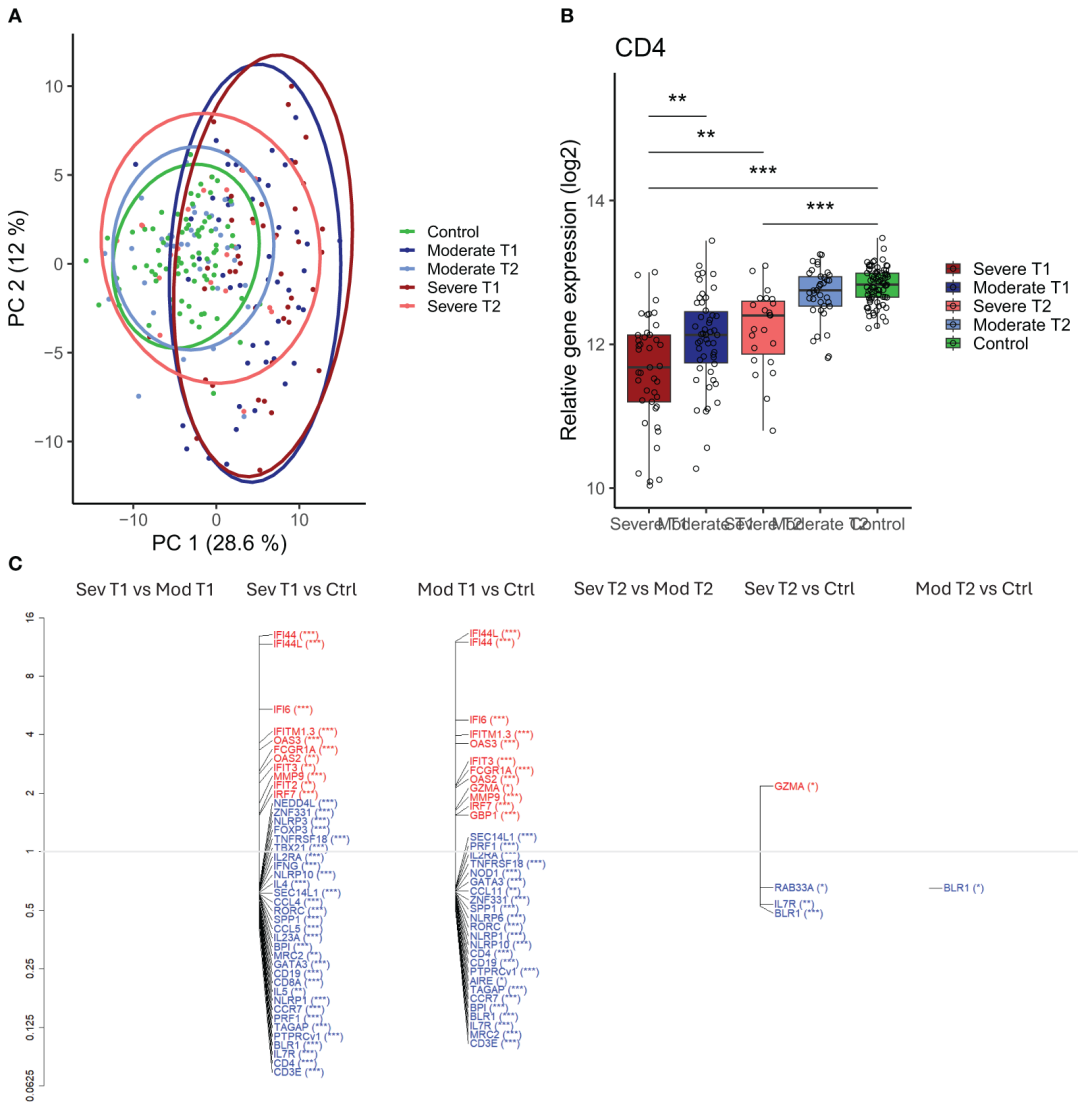
Differential gene expression in moderately and severely ill patients compared to healthy controls. **(A)** Scaled principal component analysis (PCA) plot representing individuals, status, and timepoint. **(B)** CD4 gene expression in severely and moderately ill patients at infection and convalescence and in healthy controls. Each point represents an individual, box plot shows group median with 25th and 75th percentiles, and whiskers indicate 1.5 × interquartile range (IQR). Differences were analyzed by linear regression analysis. **(C)** Differentially expressed genes (DEGs) at infection and convalescence between all participant groups. The vertical axis (y-axis) corresponds to the fold change (axis on log2 scale), and the FDR-adjusted p- values are indicated as significance levels in parentheses. Red color represents genes with higher expression, and blue color represents genes with lower expression. N = 230 (severe at infection, n = 41; moderate at infection, n = 50; severe at convalescence, n = 23; moderate at convalescence, n = 37; control, n = 79). *adj p < 0.05, **adj p < 0.01, ***adj p < 0.001. IQR, interquartile range; FDR, false discovery rate.

The original version of this article has been updated.

