# Supplementary material for: High-dimensional single-cell phenotyping unveils persistent differences in immune cell profiles between severe and moderate seasonal influenza
Source: Front Immunol. 2025 Jul 22;16:1576861. doi: 10.3389/fimmu.2025.1576861 (PMC12322504; doi:10.3389/fimmu.2025.1576861)
Supplement: Supplementary file 1 [file Table1.docx]

**High-dimensional single-cell phenotyping unveils persistent differences in immune cell profile between severe and moderate seasonal influenza - Supplementary tables and figures**

**Supplementary tables**

**Supplementary Table 1. Patient characteristics at convalescence (T2)**

| **Complete cohort** |  |  |  |  |  | **CyTOF cohort** |  |  |  |  |
| --- | --- | --- | --- | --- | --- | --- | --- | --- | --- | --- |
|  | **Controls** | **Patients** | **Mod** | **Sev** | **Sign.**  **Mod vs Sev** | **Controls** | **Patients** | **Mod** | **Sev** | **Sign. Mod vs Sev** |
| **n** | 79 | 60 | 37 | 23 | NA | 15 | 16 | 11 | 5 | NA |
| **Age, median years (range)** | 64 (32, 86) | 68 (26, 88) | 65 (26, 86) | 71 (37, 88) | NS | 66 (37, 86) | 64.5 (26, 87) | 62 (26, 83) | 80 (61, 87) | NS |
| **Females,**  **n (%)** | 55 (70) | 28 (47) | 20 (54) | 8 (35) | NS | 7 (47) | 6 (38) | 6 (55) | 0 (0) | NS |
| **Zero risk factor, n (%)** | 34 (43) | 6 (10) | 6 (16) | 0 (0) | NS | 5 (33) | 6 (38) | 5 (45) | 1 (20) | NS |
| **Two or more risk factor,**  **n (%)** | 16 (20) | 38 (63) | 21 (57) | 17 (74) | NS | 3 (20) | 6 (38) | 2 (18) | 4 (80) | NS |
| **Intensive care admission (ICU),**  **n (%)** | NA | 4 (7) | 0 (0) | 4 (17) | 0,036 | NA | 1 (6) | 0 (0) | 1 (20) | NS |
| **Receiving seasonal influenza vaccine, n (%)** | NA | 24 (40) | 14 (38) | 10 (43) | NS | NA | 6 (38) | 4 (36) | 2 (40) | NS |
| **Influenza type*, n (%)** | NA | NA | NA | NA | NA | NA | NA | NA | NA | NA |
| **B** | NA | 24 (40) | 16 (43) | 8 (35) | NS | NA | 0 (0) | 0 (0) | 0 (0) | NA |
| **A/H1N1** | NA | 5 (8) | 4 (11) | 1 (4) | NS | NA | 0 (0) | 0 (0) | 0 (0) | NA |
| **A/H3N2** | NA | 30 (50) | 16 (43) | 14 (61) | NS | NA | 16 (100) | 11 (100) | 5 (100) | NA |
| **Previous CMV infection, n (%)** | 53 (67) | 42 (70) | 26 (70) | 16 (70) | NS | 10 (67) | 9 (56) | 7 (64) | 2 (40) | NS |
| **High viral load,**  **n (%)** | NA | NA | NA | NA | NA | NA | 12 (75) | 8 (73) | 4 (80) | NS |
| **Time from hospitaliz-ation, median days (range)** | NA | 250.5 (149, 321) | 242 (154, 308) | 253 (149, 321) | NS | NA | 262 (193, 292) | 260 (193, 292) | 267 (235, 280) | NS |

*Influenza type was defined by PCR and not detected at T2

Mod = Moderately ill influenza patients

Sev = Severely ill influenza patients

**Supplementary Table 2. Antibody panels for CyTOF analysis, unstimulated cells**

| **Panel 1: PBMC unstimulated cells (extracellular markers)** | | | | | | | |
| --- | --- | --- | --- | --- | --- | --- | --- |
| **Metal label** | **Specificity** | **Clone** | **Product number** | **Producer** | **Inhouse conj.^#^** | **Dilution** | **Staining group*** |
| y89 | CD45 | HI30 | 3089003B | Fluidigm |  | 1:200 | 3 |
| 106Cd | CD57 | HNK-1 | 359602 | BioLegend | X | 1:200 | 3 |
| 111Cd | CD19 | HIB19 | 302247 | BioLegend | X | 1:200 | 3 |
| 112Cd | CD5 | UCHT2 | 300627 | BioLegend | X | 1:100 | 3 |
| 113Cd | CD8 | SK1 | 344727 | BioLegend | X | 1:200 | 3 |
| 114Cd | HLA-DR | L243 | 307651 | BioLegend | X | 1:200 | 3 |
| 116Cd | CD3 | UCHT1 | 300443 | BioLegend | X | 1:200 | 3 |
| 141Pr | CCR6 | 11A9 | 3141014A | Fluidigm |  | 1:50 | 1 |
| 142Nd | KLRG1 | 13F12F2 | 16948885 | Invitrogen | X | 1:200 | 1 |
| 143Nd | CD127 | A019D5 | 3143012B | Fluidigm |  | 1:100 | 2 |
| 144Nd | CD15 | W6D3 | 3144019B | Fluidigm |  | 1:100 | 3 |
| 145Nd | CD4 | RPA-T4 | 3145001B | Fluidigm |  | 1:100 | 3 |
| 146Nd | IgD | IA-62 | 3146005B | Fluidigm |  | 1:200 | 3 |
| 147Sm | CD11c | Bu15 | 3147008B | Fluidigm |  | 1:100 | 2 |
| 148Nd | CD16 | 3G8 | 3148004B | Fluidigm |  | 1:100 | 3 |
| 149Sm | CD25 | 2A3 | 3149010B | Fluidigm |  | 1:100 | 2 |
| 150Nd | CD134/OX40 | ACT35 | 3150023B | Fluidigm |  | 1:100 | 2 |
| 151Eu | CD123 | 6H6 | 3151001B | Fluidigm |  | 1:100 | 3 |
| 152Sm | TCRγδ | 11F2 | 3152008B | Fluidigm |  | 1:100 | 2 |
| 153Eu | CCR4/CD194 | L291H4 | 3153030A | Fluidigm |  | 1:100 | 1 |
| 154Sm | TIGIT | MBSA43 | 3154016B | Fluidigm |  | 1:50 | 1 |
| 155Gd | CD45RA | HI100 | 3155011B | Fluidigm |  | 1:200 | 2 |
| 156Gd | CXCR3/CD183 | G025H7 | 3156004B | Fluidigm |  | 1:100 | 1 |
| 158Gd | CD27 | L128 | 3158010B | Fluidigm |  | 1:200 | 3 |
| 159Tb | IgG | G18-145 | 555784 | BD biosciences | X | 1:100 | 2 |
| 160Gd | CD28 | CD28.2 | 3160003B | Fluidigm |  | 1:200 | 2 |
| 161Dy | CD160 | 688327 | MAB6700 | R&D | X | 1:100 | 1 |
| 162Dy | CD95 | DX2 | 3162038B | Fluidigm |  | 1:100 | 2 |
| 163Dy | CRTH2/CD294 | BM16 | 3163003B | Fluidigm |  | 1:100 | 1 |
| 164Dy | CD161 | HP-3G10 | 3164009B | Fluidigm |  | 1:100 | 1 |
| 165Ho | CD85j | GHI/75 | 333721 | BioLegend | X | 1:100 | 2 |
| 166Er | TCRVa7.2 | 3C10 | 351702 | BioLegend | X | 1:100 | 2 |
| 167Er | CCR7/CD197 | G043H7 | 3167009A | Fluidigm |  | 1:100 | 1 |
| 168Er | ICOS/CD278 | C398.4A | 313539 | BioLegend | X | 1:100 | 2 |
| 169Tm | NKG2A | Z199 | 3169013B | Fluidigm |  | 1:100 | 2 |
| 170Er | CD169 | 7-239 | 346002 | BioLegend | X | 1:50 | 2 |
| 171Yb | CXCR5/CD185 | RF8B2 | 3171014B | Fluidigm |  | 1:100 | 1 |
| 172Yb | CD38 | HIT2 | 3172007B | Fluidigm |  | 1:100 | 3 |
| 173Yb | CD141 | 1A4 | 3173002B | Fluidigm |  | 1:50 | 3 |
| 174Yb | CD279-PD-1 | EH12.2H7 | 3174020B | Fluidigm |  | 1:50 | 1 |
| 175Lu | CD14 | M5E2 | 3175015B | Fluidigm |  | 1:50 | 3 |
| 176Yb | CD56 | NCAM16.2 | 3176008B | Fluidigm |  | 1:100 | 3 |
| 209Bi | CD11b | ICRF44 | 3209003B | Fluidigm |  | 1:200 | 3 |
| 194Pt | Cisplatin - live/dead |  | 201194 | Fluidigm |  | 1:1000 | Viability |
| 191Ir | Cell-ID Intercalator |  | 201192B | Fluidigm |  | 1:4000 | Intercalator |
| 193Ir | Cell-ID Intercalator |  | 201192B | Fluidigm |  | 1:4000 | Intercalator |

^#^Inhouse conjugations with MaxPar Antibody Labeling Kits following manufacturer’s instructions

*Explanations for the staining group conditions in Supplementary Table 6.

**Supplementary Table 3. Overview of how markers are used for cell subpopulation annotation in the CyTOF panel.**

| **Cellpopulation** | **Subpopulation** | **Markers** |
| --- | --- | --- |
| **T cytotoxic** |  | **CD3, CD8** |
|  | Tc naive | CD45RA, CCR7 |
|  | Tc central memory | CD45RAneg CCR7 |
|  | Tc effector | CD45RA, CCR7neg, KLRG1, CD160 |
|  | Tc effector memory | CD45RAneg, CCR7neg, KLRG1, CD160 |
| **T helper** |  | **CD3, CD4** |
|  | Th naive | CXCR3, CD45RA, CCR7, CD28, CD95neg |
|  | Th central memory | CXCR3, CD45RAneg, CCR7, CD95 |
|  | Th effector | CXCR3, CD45RA, CCR7neg |
|  | Th effector memory | CXCR3, CD45RAneg, CCR7neg |
| **Th1** |  | **CD3, CD4, CXCR3** |
| **Th2** |  | **CD3, CD4, CCR4** |
| **Th17** |  | **CXCR3neg, CCR6, *IL-17*** |
| **Treg** |  | **CD3, CD4, CD25, FoxP3, CD127low, CCR4** |
|  | Activated Treg | HLA-DR, CTL-A |
|  | Naive Treg | CD28, CD45RA |
| **T follicular** |  | **CXCR5, ICOS** |
|  | Tfh | ICOS, PD-1 |
| **δγT-cell** |  | **CD3, TCR**δγ |
| **MAIT** |  | **CD3, TCRVA7.2** |
| **NKT** | immature | **CD3, CD56**  CD56bright |
|  | mature | CD56dim, CD16, CD57+/- |
| **NK** | precursor | **CD3neg, CD56CD161**, **CD160**  CD3neg, CD56neg, CD160, CD161 |
|  | immature | CD3neg, CD56bright, CD161 |
|  | mature | CD3neg, CD56dim, CD161 |
| **B** |  | **CD19, HLA-DR** |
|  | naive | CD19, IgD, CD28, CD27neg, CD127 |
|  | memory | CD27, IgD+/- |
|  | plasmablast | CD38, CD19neg |
| **DC** |  | **CD45, HLA-DR**, CD11c |
|  | plasmacytoid | CD123 |
|  | myeloid DC | CD11c, CD14 1, HLA-DR |
|  | immature | CD127 |
| **Monocyte** |  | **CD169***,* CD33, CD14; CD16, CD11b, CD141 |
|  | classical | CD33, CD14high, CD16neg |
|  | non classical | CD33, CD14, CD16 |
| **Neutrophil** |  | CD15 |
| **Granulocyte** |  | CD11b |
|  |  |  |
| **Functional markers** | Exhaustion | PD-1 (T, NK) |
|  | Checkpoint -inhibitory | CD85j (NK, T, Mo) |
|  | Checkpoint -inhibitory | LAG-3 (T, NK) |
|  | Checkpoint -inhibitory | CTLA-4/CD152 (Tregs) |
|  | Checkpoint -inhibitory  Checkpoint -inhibitory | NKG2A (NK)  CD5 (B, T) |
|  | Checkpoint -apoptosis | CD95 (T,B, monocytes, neutrophils) |
|  | Checkpoint -inhibitory/activating | TIM-3 activating/inactivating (T, NK) |
|  | Checkpoint -activating | CD134/OX40 (Tregs, T) |
|  | Checkpoint -activating | TIGIT (Tregs, NK) |
|  | Checkpoint -activating | CRTH2/CD294 (Th2, eosinophils, basophils) |
|  |  |  |
|  | Cytokines | *IL-1b* |
|  |  | *IL-2* |
|  |  | *IL-6* |
|  |  | *IL-10* |
|  |  | *IL-12p70* |
|  |  | *IL-17a* |
|  |  | *IFNg* |
|  |  | *TNFa* |
|  |  | *Perforin* |
|  |  | *GranzymeB* |
|  |  |  |

**Supplementary Table 4. Supervised gating of cell populations**

| **Cell population** | **Markers** |
| --- | --- |
| CD4 | CD45, CD3, CD4, CD19neg, CD8neg, TCRγδneg, TCRVa7.2neg, CD8neg |
| CD8 | CD45, CD3, CD8, CD19neg, CD4neg |
| gdT | CD45, CD3, TCRγδ, TCRVa7.2neg, CD19neg |
| MAIT | CD45, CD3, TCRγδneg, TCRVa7.2, CD19neg |
| B | CD45, CD3neg, CD19, CD56neg, HLA-DR |
| NK | CD45, CD3neg, CD19neg, CD56, TCRγδneg; CD57high if CD56low |
| NKT | CD45, CD3, CD19neg, CD56high/low, TCRγδneg; CD57high if CD56high |
| Mo | CD45, CD3neg, CD19neg, CD56neg, CD57neg, CD14high/low; CD14neg if CD38neg; CD169 |
| DC | CD45, CD3neg, CD19neg, CD56neg, CD57neg, HLA-DRhigh/low, CD169neg, CD11cneg, CD11b |

**Supplementary Table 5. Antibody panels for CyTOF analysis, stimulated cells**

| **Panel 2: PBMC stimulated cells (extra and intracellular markers)** | | | | | | | |
| --- | --- | --- | --- | --- | --- | --- | --- |
| **Metal label** | **Specificity** | **Clone** | **Product #** | **Producer** | **Inhouse conj.^#^** | **Dilution** | **Staining group *** |
| y89 | CD45 | HI30 | 3089003B | Fluidigm |  | 1:200 | 3 |
| 106Cd | CD57 | HNK-1 | 359602 | BioLegend | X | 1:200 | 3 |
| 110Cd | CD107a | H4A3 | 328635 | BioLegend | X | 1:100 | 0 |
| 111Cd | CD19 | HIB19 | 302247 | BioLegend | X | 1:200 | 3 |
| 112Cd | CD44 | BJ18 | 338811 | BioLegend | X | 1:200 | 2 |
| 113Cd | CD8 | SK1 | 344727 | BioLegend | X | 1:200 | 3 |
| 114Cd | HLA-DR | L243 | 307651 | BioLegend | X | 1:200 | 3 |
| 116Cd | CD3 | UCHT1 | 300443 | BioLegend | X | 1:200 | 3 |
| 141Pr | CD223/LAG3 | 11C3C65 | 369302 | BioLegend | X | 1:50 | 1 |
| 142Nd | IL-1b | H1b-27 | 511602 | BioLegend | X | 1:50 | 4 |
| 143Nd | CD127/IL-7Ra | A019D5 | 3143012B | Fluidigm |  | 1:100 | 2 |
| 144Nd | IL-2 | MQ1-17H12 | 3144021B | Fluidigm |  | 1:100 | 4 |
| 145Nd | CD4 | RPA-T4 | 3145001B | Fluidigm |  | 1:100 | 4 |
| 146Nd | TNFa | Mab11 | 3146010B | Fluidigm |  | 1:200 | 4 |
| 147Sm | TIM-3 | F38-2E2 | 345019 | BioLegend | X | 1:50 | 1 |
| 148Nd | CD274/PD-L1 | 29E.2A3 | 3148017B | Fluidigm |  | 1:50 | 2 |
| 149Sm | IL-12p70 | 7B12 | 511005 | BioLegend | X | 1:50 | 4 |
| 150Nd | MIP-1b | D21-1351 | 3150004B | Fluidigm |  | 1:100 | 4 |
| 151Eu | CD137/4-1BB | 4B4-1 | 309841 | BioLegend | X | 1:50 | 2 |
| 152Sm | TCRγδ | 11F2 | 3152008B | Fluidigm |  | 1:100 | 2 |
| 153Eu | CXCR5/CD185 | RF8B2 | 3153020B | Fluidigm |  | 1:100 | 1 |
| 154Sm | CD272/BTLA | MIH26 | 344529 | BioLegend | X | 1:50 | 1 |
| 155Gd | CD45RA | HI100 | 3155011B | Fluidigm |  | 1:200 | 2 |
| 156Gd | IL-6 | MQ2-13AS | 3156011B | Fluidigm |  | 1:100 | 4 |
| 158Gd | CD27 | L128 | 3158010B | Fluidigm |  | 1:200 | 3 |
| 159Tb | GM-CSF | BVD2-21C11 | 3159008B | Fluidigm |  | 1:100 | 4 |
| 160Gd | CD28 | CD28.2 | 3160003B | Fluidigm |  | 1:200 | 2 |
| 161Dy | IL-17A | BL168 | 3161008B | Fluidigm |  | 1:100 | 4 |
| 162Dy | FoxP3 | PCH101 | 3162011A | Fluidigm |  | 1:100 | 4 |
| 163Dy | CD33 | WM53 | 3163023B | Fluidigm |  | 1:50 | 3 |
| 164Dy | Perforin | dG9 | 308102 | BioLegend | X | 1:50 | 4 |
| 165Ho | IFNg | B27 | 3165002B | Fluidigm |  | 1:100 | 4 |
| 166Er | IL-10 | JES3-9D7 | 3166008B | Fluidigm |  | 1:100 | 4 |
| 167Er | CCR7 /CD197 | G043H7 | 3167009A | Fluidigm |  | 1:100 | 1 |
| 168Er | CD154/CD40L | 24-31 | 3168006B | Fluidigm |  | 1:50 | 2 |
| 169Tm | CD25 | 2A3 | 3169003B | Fluidigm |  | 1:100 | 2 |
| 170Er | CTLA-4/CD152 | 14D3 | 3170005B | Fluidigm |  | 1:50 | 4 |
| 171Yb | Granzyme B | GB11 | 3171002B | Fluidigm |  | 1:200 | 4 |
| 172Yb | CD38 | HIT2 | 3172007B | Fluidigm |  | 1:100 | 3 |
| 173Yb | CD273/PD-L2 | 24F.10C12 | 329613 | BioLegend | X | 1:50 | 2 |
| 174Yb | CD279/PD-1 | EH12.2H7 | 3174020B | Fluidigm |  | 1:50 | 1 |
| 175Lu | CD14 | M5E2 | 3175015B | Fluidigm |  | 1:50 | 3 |
| 176Yb | CD56 | NCAM16.2 | 3176008B | Fluidigm |  | 1:100 | 3 |
| 209Bi | CD16 | 3G8 | 3209002B | Fluidigm |  | 1:100 | 3 |
| 194Pt | Cisplatin - live/dead |  | 201194 | Fluidigm |  | 1:1000 | Viability |
| 191Ir | Cell-ID Intercalator |  | 201192B | Fluidigm |  | 1:4000 | Intercalator |
| 193Ir | Cell-ID Intercalator |  | 201192B | Fluidigm |  | 1:4000 | Intercalator |

^#^Inhouse conjugations were done using MaxPar Antibody Labeling Kits following manufacturer’s instructions

*Explanations for the staining group conditions in Supplementary Table 6.

**Supplementary Table 6. Preparation and staining of cells for CyTOF analyses.**

| **Staining groups** | **Incub. minutes** | **Temp.** |
| --- | --- | --- |
| **0** During stimulation (only CD107a) | 4 hours | 37°C |
| **FC-block** (Human TruStain) | 10 | 37°C |
| **1** (chemokine receptors, low ab staining) ^¤^ | 10 | 37°C |
| **2** (intermediate ab staining) ^¤^ | 20 | RT |
| **3** (strong ab staining) ^¤^ | 30 | on ice |
| **Viability** (Cisplatin 194 Pt) | 5 | RT |
| **4** (intracellular markers, in Panel 2)***** | 40 | on ice |
| **Intercalator** (Cell-ID) | 20 | RT |
| **Fixation and permeabilisation** |  |  |
| **Panel 1 - Fresh fix**, 1,6% Formaldehyde*  (after viability staining) | 10 | RT |
| **Panel 1 - 1X Fix and Perm buffer**** (together with intercalator) | 20 | RT |
| **Panel 2 - 1X Fix 1 buffer***** (after viability staining) | 20 | RT |
| **Panel 2 - Permeabilisation**, ice cold (-20°C) MeOH****  (after Fix 1 buffer) | approx. 5 weeks | -80°C |
| **Panel 2 - 1X PermS buffer******* (together with staining group 4) | 40 | on ice |
| **Panel 2 - Fresh fix**, 1,6% Formaldehyde* (after staining group 4)) | 10 | RT |
| **Panel 2 - 1X Fix and Perm buffer**** (together with intercalator) | 20 | RT |

**Ab:** antibodies

^¤^ Maxpar® Cell Staining Solution, Fluidigm # 201068

* 1:10 in PBS of 16% Formaldehyde, methanol free (Pierce,Thermo Fisher Sci. #28906)

** Maxpar® Fix and Perm Buffer, Fluidigm #201067

*** 1:5 in PBS of Maxpar® Fix I Buffer, Fluidigm #201065

**** Metanol ≥99.8%, AnalaR NORMAPUR® ACS, Reag. Ph. Eur. Analytic reagent, VWR/Avantor #20847.307

***** Maxpar® Perm-S Buffer, Fluidigm #201066

**Supplementary Table 7. Genes and targets included in the gene expression analyses.**

| **Module** | **Gene** |
| --- | --- |
| Immune cell subset markers - B cells | CD19 |
| NK cells | NCAM1 |
| T cell subset markers | CD3E, CD4, CD8A, CCR7, PTPRCv1, PTPRCv2, AIRE, IL7R |
| Th1 associated genes | CXCL10, IFNG, IL1B, IL2, IL15, TBX21, TNF |
| Th2 associated genes | GATA3, IL4, IL4δ2, IL5, IL6, IL10, IL13 |
| Th9 associated genes | IL9 |
| Th17 associated genes | IL17A, RORC, IL22RA1 |
| Treg associated genes | CCL4, CTLA4, FOXP3, IL2RA, LAG3, TGFB1, TNFRSF18 |
| Cytotoxicity markers | GNLY, GZMA, GZMB, PRF1 |
| Apoptosis / Survival | CASP8, BCL2, FASLG, FLCN1, TNFRSF1A, TNFRSF1B |
| Myeloid associated genes | CD14, CD163, CCL2, CCL3, CCL5, CCL22, CXCL13, IL12A, IL12B, FPR1 |
| Chemokines | CCL11, CCL13, CCL19, CXCL9, CX3CL1 |
| Pattern recognition receptors | CD209, CLEC7A, MRC1, MRC2, NOD1, NOD2, TLR1, TLR2, TLR3, TLR4, TLR5, TLR6, TL7, TLR8, TLR9, TLR10 |
| Inflammasome components | NLRC4, NLRP1, NLRP2, NLRP3, NLRP4, NLRP6, NLRP7, NLRP10, NLRP11, NLRP12, NLRP13 |
| IFN signaling genes | CD274, FCGR1A, GBP1, GBP2, GBP5, IFI6, IFI16, IFI35, IFI44, IFI44L, IFIH1, IFIT2, IFIT3, IFIT5, IFITM1/3, INDO, IRF7, OAS1, OAS2, OAS3, SOCS1, STAT1, STAT2, TAP1, TAP2 |
| Inflammation | DSE, MMP9, SPP1, TIMP2, TNIP1 |
| Cell growth / Proliferation | BMP6, TGFBR2, AREG, EGF, VEGF |
| Cell activation | HCK, LYN, SLAMF7 |
| Small GTPases / (Rho) GTPase activating proteins | ASAP1, RAB13, RAB24, RAB33A, TAGAP, TBC1D7 |
| Anti-microbial activity | BPI, LTF |
| E3 ubiquitin protein ligases | NEDD4L |
| Scavenger receptors | MARCO |
| G protein-coupled receptors | BLR1 |
| Transcriptional regulators/activators | CAMTA1, TWIST1, ZNF331, ZNF532 |
| Intracellular transport | SEC14L1, KIF1B |
| Mitochondrial Stress / Proteasome | HPRT |
| Housekeeping | ABR, B2M, GAPDH, GUSB |

**Supplementary figures**

**Supplementary Figure 1**


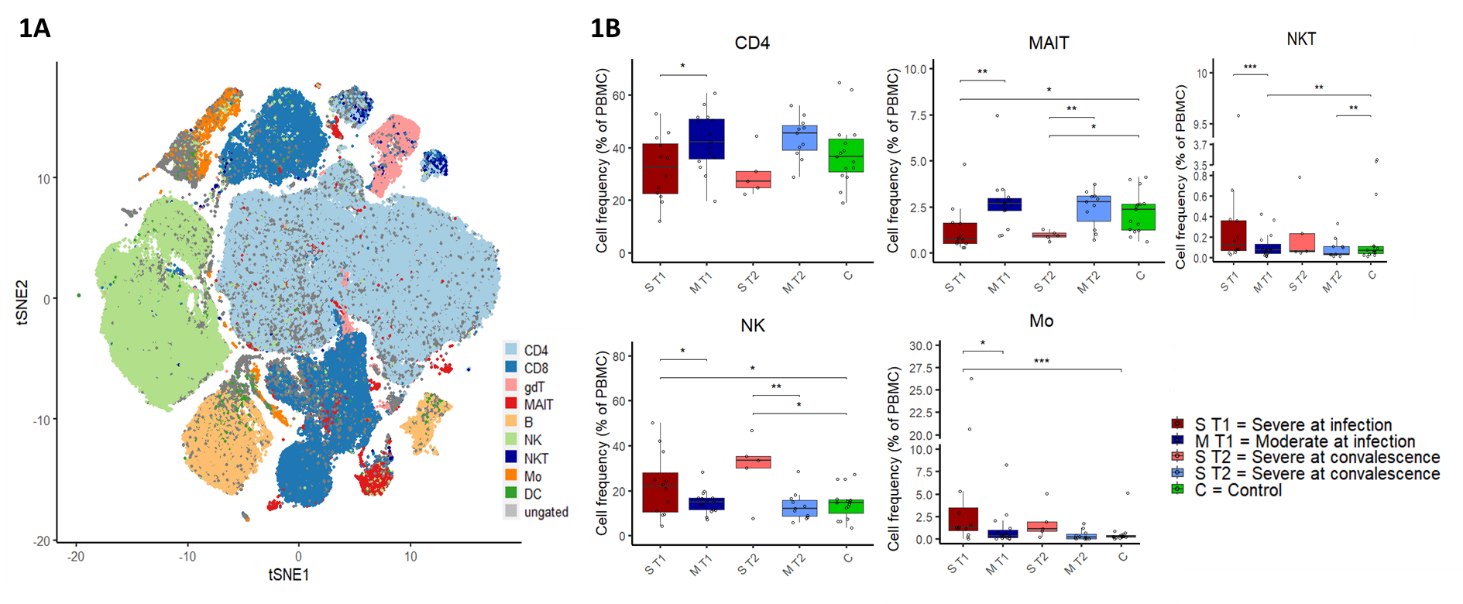


**Figure 1. Differences in cell population frequencies between severe and moderate influenza patients at T1, supervised analysis. A)** tSNE plot visualizing the cell distribution based on expression of all markers on cells from all samples at both time points (n=58) colored/ by the nine major populations identified by the supervised gating strategy in supplementary Table 4. **B)** Percentages of the five cell populations that were significantly different between severely and moderately ill patients at T1 based on negative binomial regression analysis, shown for all groups and time points. ST1 = severely ill patients at infection, T1 (n = 12), MT1 = moderately ill patients at T1 (n = 15), ST2 = severe at convalescence, T2 (n = 5), MT2 = moderate at T2 (n = 11) and C = healthy control (n = 15). Dots represent a single participant while the box indicates median with 25-75 percentile, the whiskers indicate the 1.5 x interquartile range (IQR) range. The p-values are FDR-adjusted comparing the nine manually gated cell populations (*adj.p <0.05, **adj.p<0.01, ***adj.p<0.001).

**Supplementary Figure 2**

**
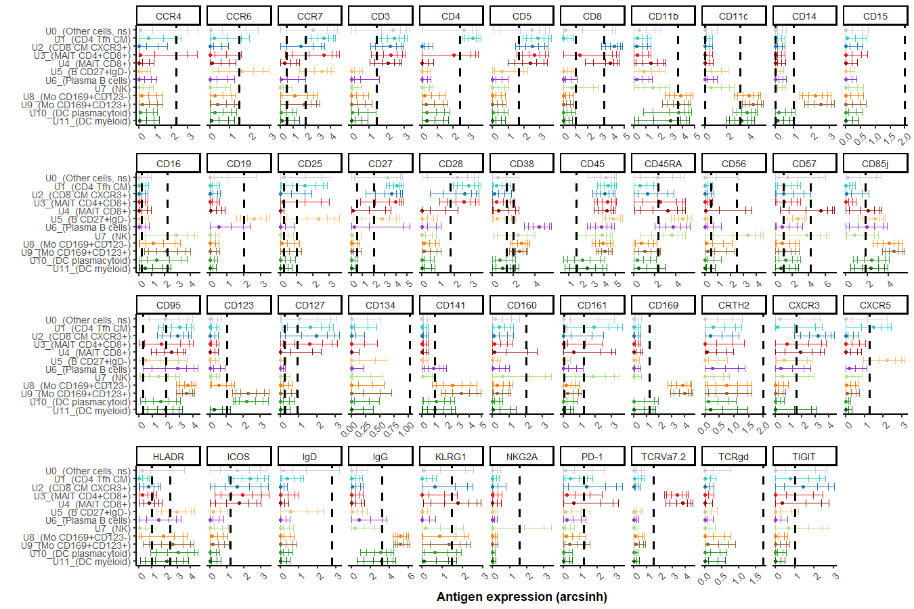
**
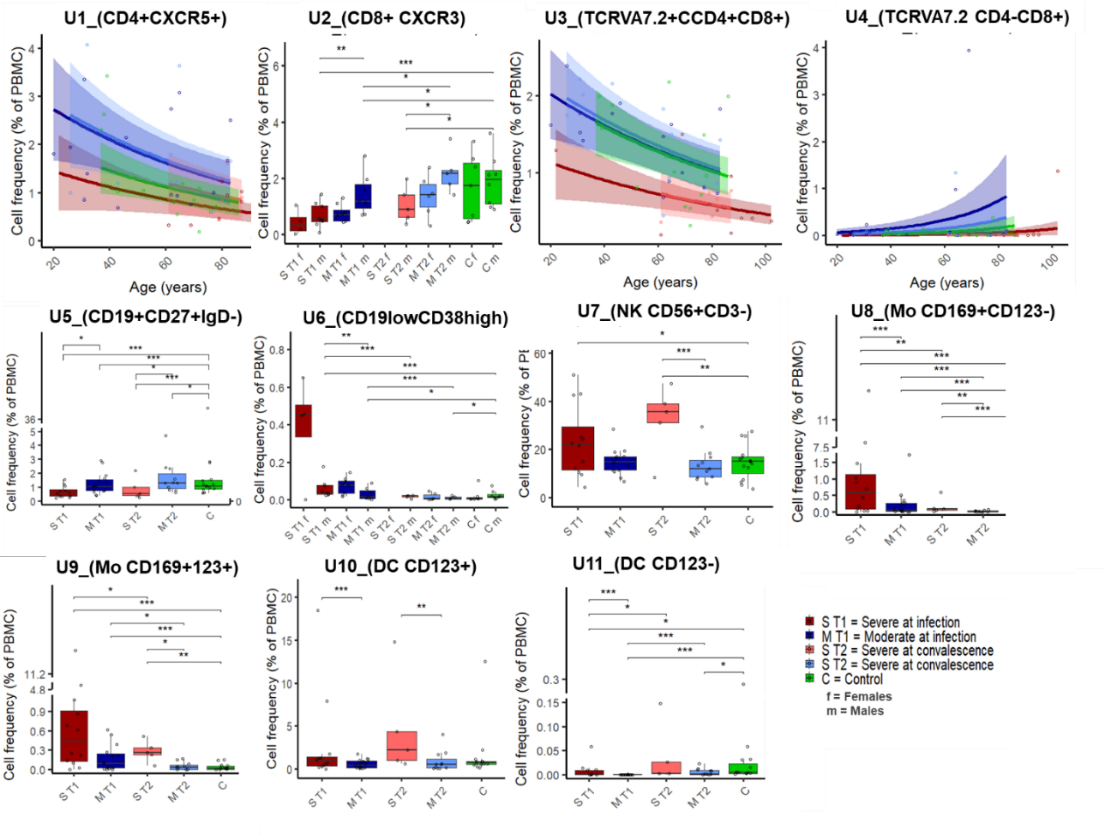
**
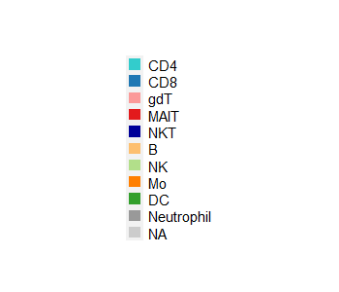
A** **B C**


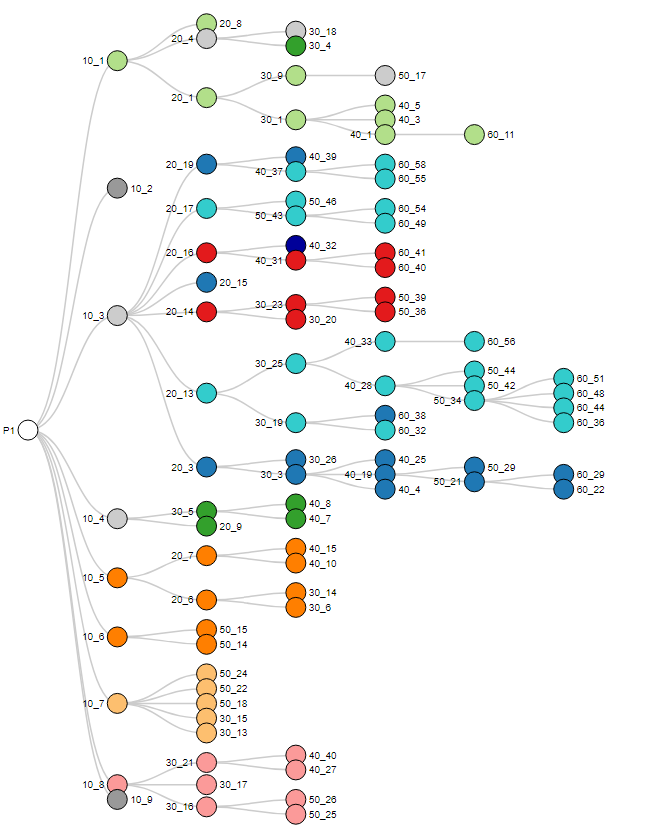


**D**

**
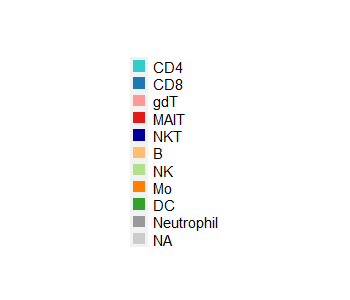
**
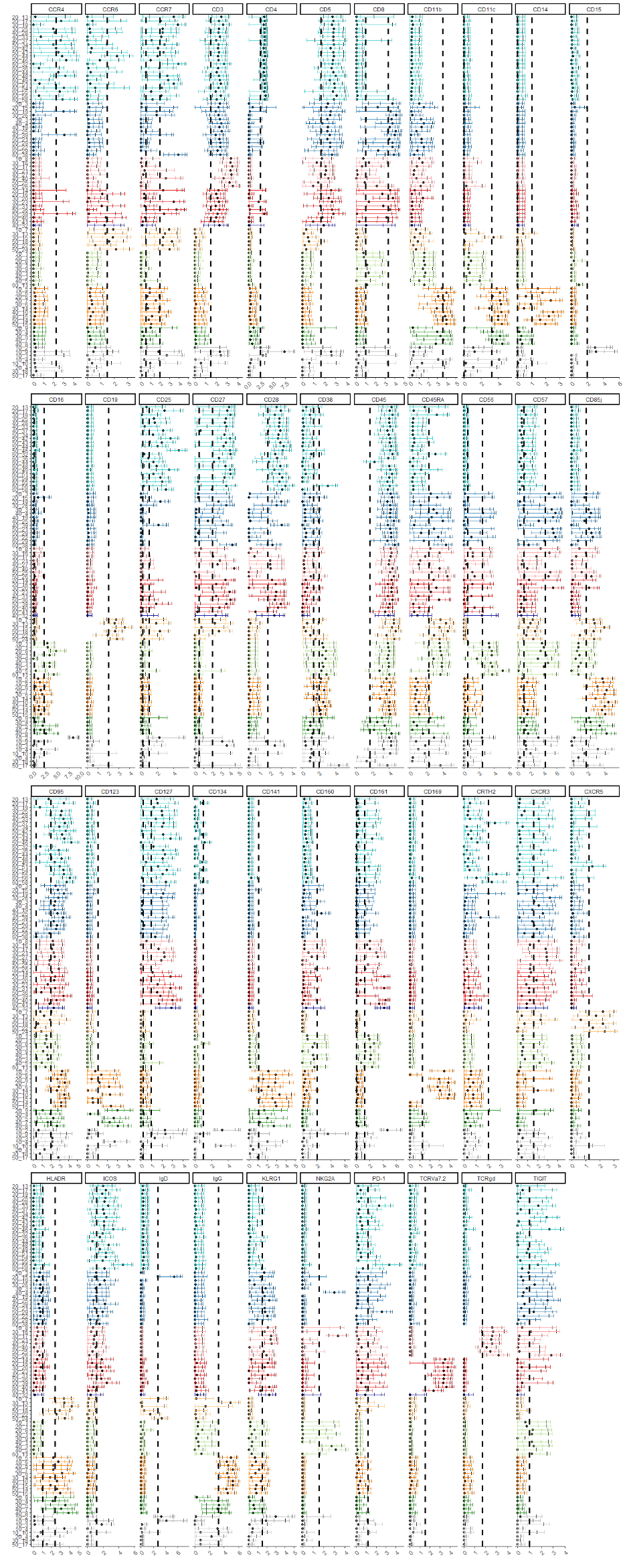


**Supplementary Figure 2. Differential cell frequencies in severely and moderately ill patients at infection (T1), unstimulated cells.**  **A)** Hierarchy tree of all 92 unique clusters colored by major cell type according to marker expression. The 11 clusters significantly different upon severity are marked with thick lining. **B)** Marker plot for cell characterization of the eleven clusters (Cl1-11) significantly different between severe and moderate disease at infection, T1. **C)** Cell frequency distributions of the significant clusters after manual gating of all cells using the markers identified by unsupervised clustering (ST1 = severe patients at infection, n = 12, MT1 = moderate at infection, n = 15, ST2 = severe at convalescence, n = 5, MT2 = moderate at convalescence, n = 11, C = control, n = 15). Dependency of age and sex was tested and defined the plot type. For clusters 1-11, adj p-values = *p< 0.05, **<0.01, ***<0.001. **D)** Identification of all clusters by marker expression. Horizontal bars represent 5, 10, 25, 50 (dot), 75, 90, 95 quantiles, vertical lines indicate negative/positive signal and low/high signals. Colors represent cell types based on signal intensity of phenotyping markers.

**Supplementary Figure 3**

**A**

**
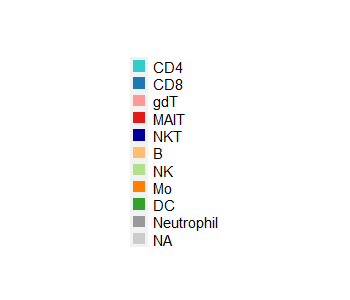

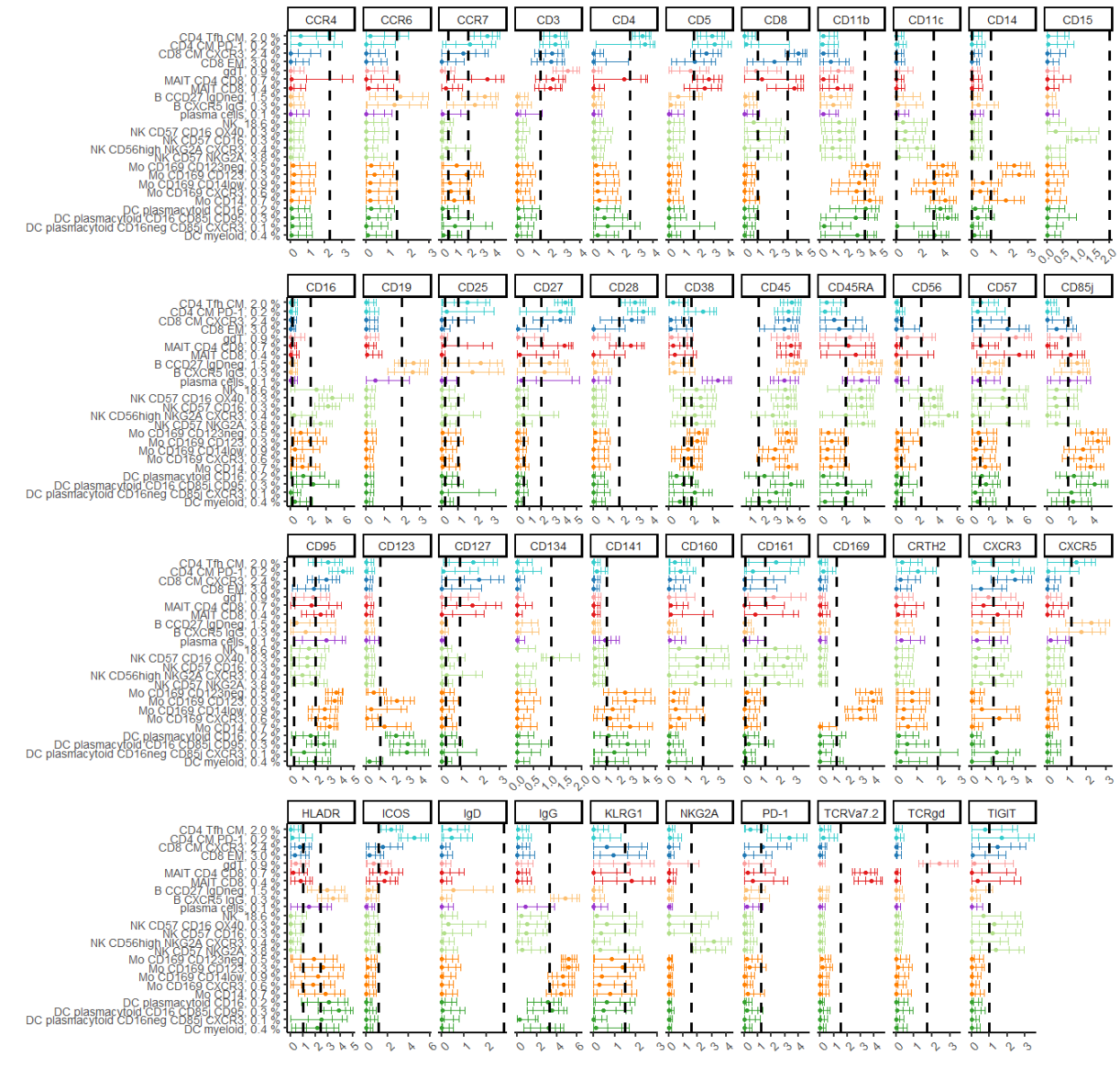
**

**B**

**
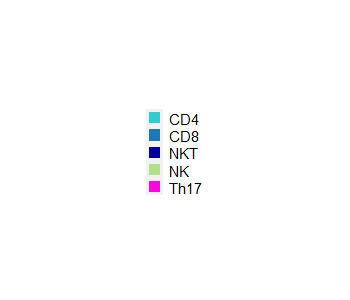

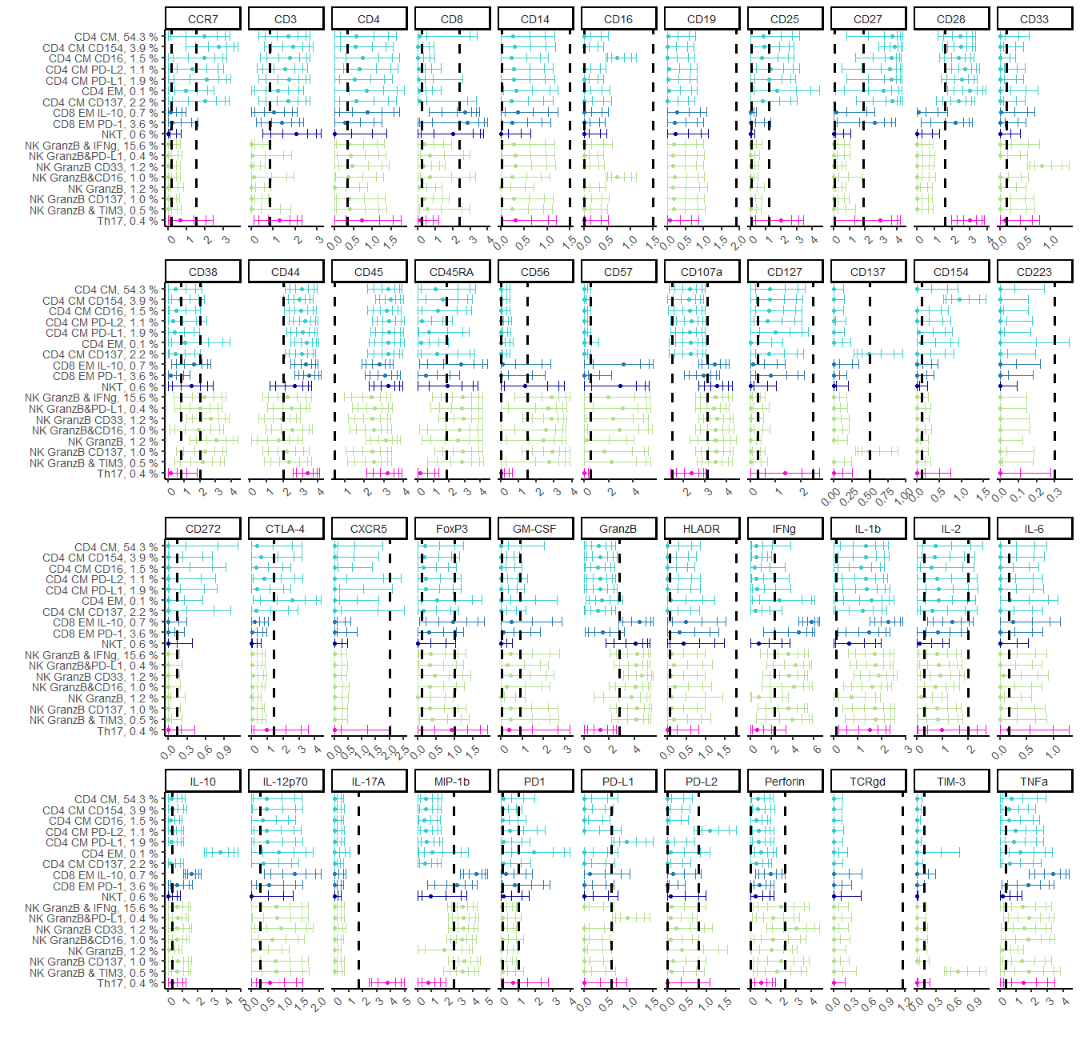
**

**Supplementary Figure 3. Marker plots of all the marker signals for the clusters significantly different between all participant groups and timepoints. A)** Marker plot of significant clusters from all analyses of unstimulated cells and **B)** stimulated cells with tentative names given based on the expressed markers. Significant clusters from the analyses: Severe T1-Moderate T1, SevT1-Control, ModT1-Control, SevT2-ModT2, SevT2-Control, ModT2-Control. Horizontal bars represent 5, 10, 25, 50 (dot), 75, 90, 95 quantiles, vertical dotted lines indicate level for distinguishing negative/positive signal and for some markers also low/high signals. Color coding represents cell type identified based on the distribution of the signal intensity of phenotyping markers.

**Supplementary Figure 4**

**
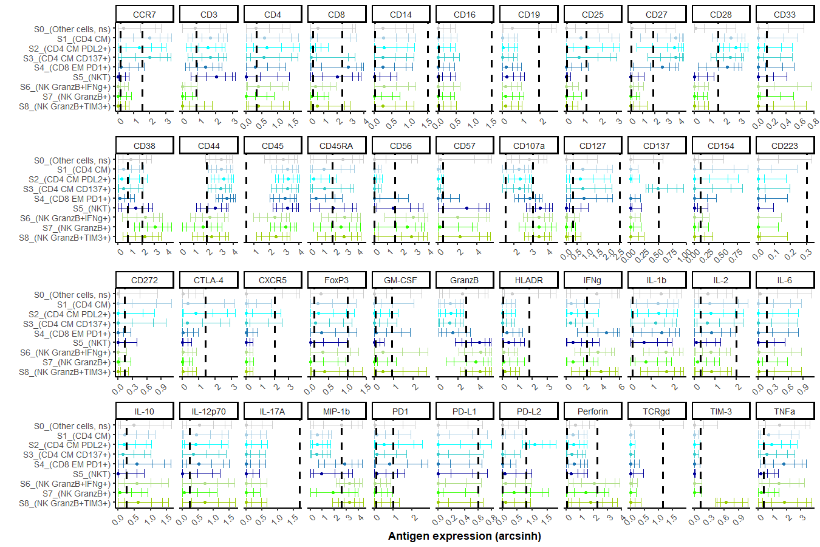

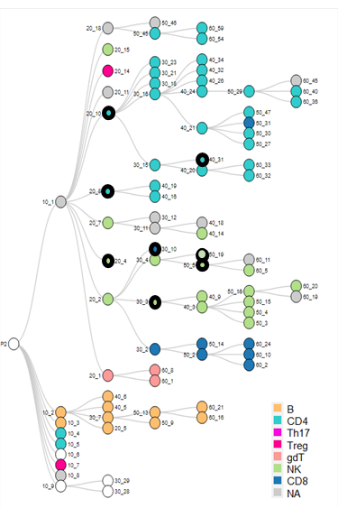
A B**

**C**

**
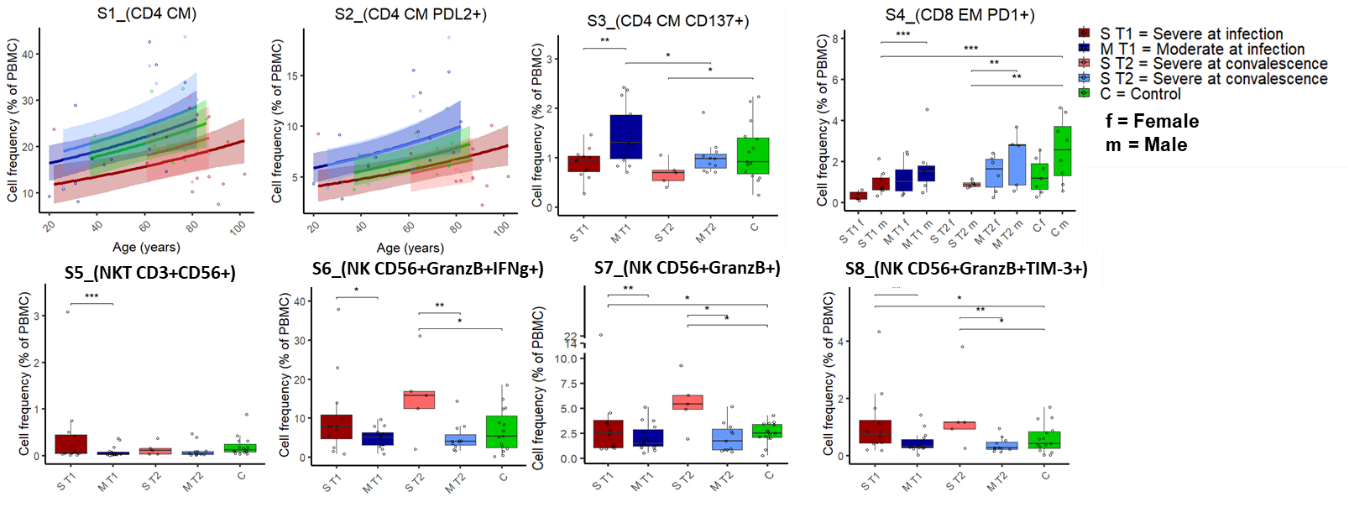
**

**D**

**
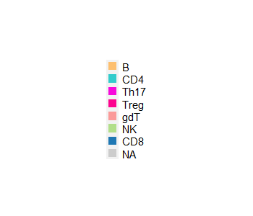

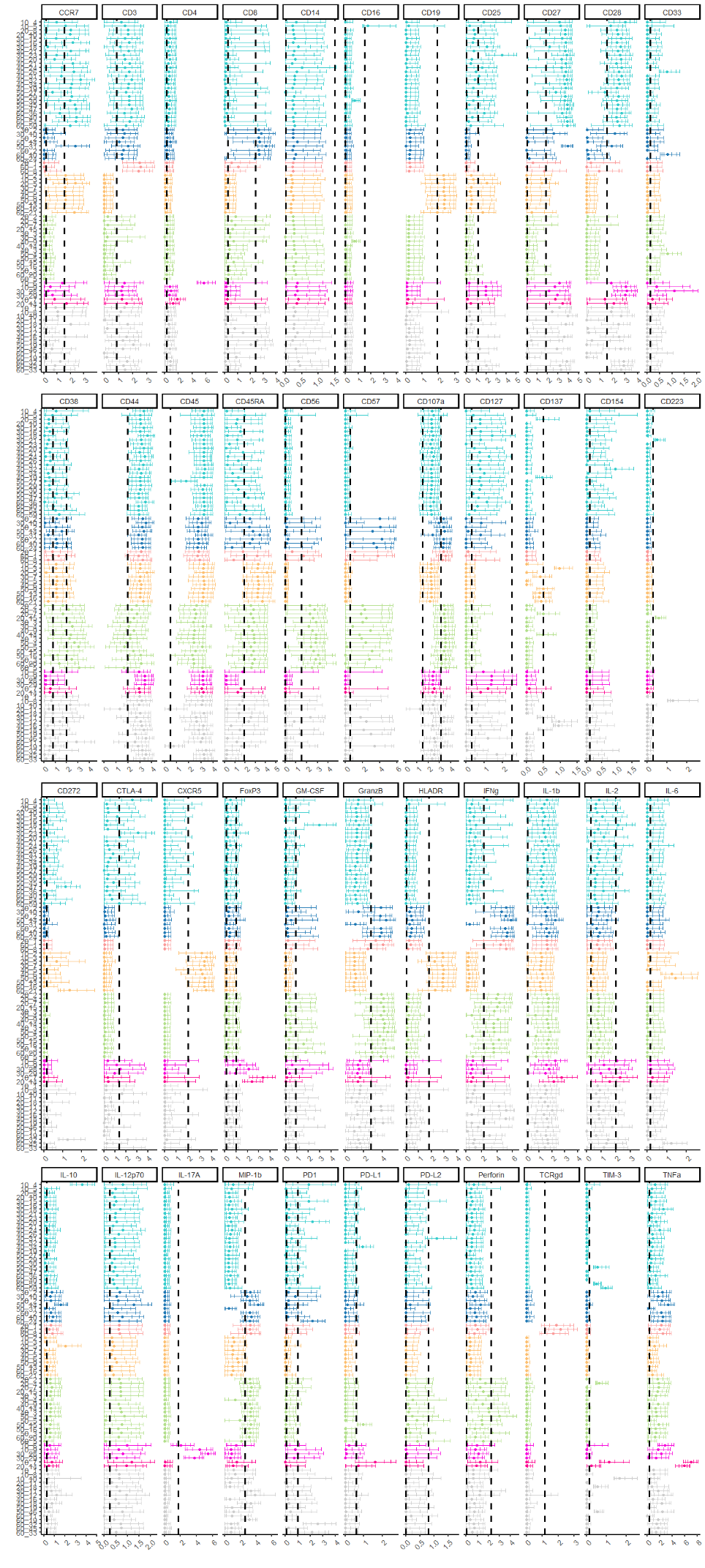
**

**Supplementary Figure 4. Differential cell frequencies in severely and moderately ill patients at T1, stimulated cells. A)** Hierarchy tree of all 85 unique clusters colored by major cell type according to major marker expression. The 8 clusters significantly different between severely and moderately ill patients are marked with thick lining. **B)** Marker plot characterizes the cells within the eight clusters (Cl1-8) significantly different between severe and moderately ill patients at infection. **C)** Cell frequency distributions of the significant clusters based on manual gating in R with the identified markers and negative binomial regression analysis (ST1 = severe at infection, n = 12, MT1 = moderate at infection, n = 15, ST2 = severe at convalescence, n = 5, MT2 = moderate at convalescence, n = 11, C = control, n = 15). Dependency of age and sex was tested and defined the plot type. For clusters 1-11, adj p-values = *p< 0.05, **<0.01, ***<0.001. **D)** Eighty-five unique clusters were identified. Horizontal bars represent 5, 10, 25, 50 (dot), 75, 90, 95 quantiles, vertical dotted lines indicate level for distinguishing negative/positive signal and low/high signals. Colors represent cell types identified based on phenotyping markers.

**Supplementary Figure 5**


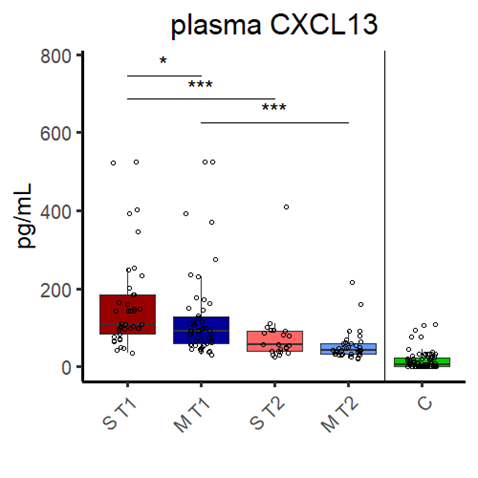
**Supplementary Figure 5. Plasma CXCL13 levels in all participants**. The vertical line in this plot indicates difference in plasma collection and controls are therefore not included in the Wilcoxon rank sum test. Severe T1 (n=41), Moderate T1 (n=50), Severe T2 (n=23), Moderate T2 (n=37), Control (n=79), in total n=230). *p<0.05, ***p<0.001.

**Supplementary Figure 6**


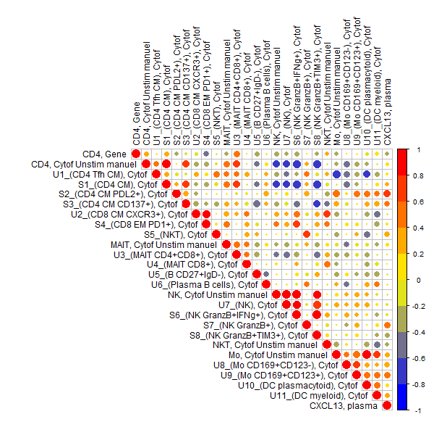

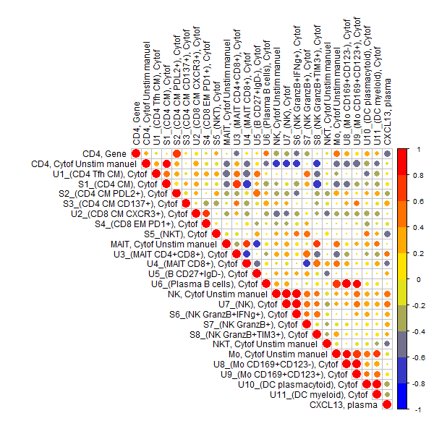


**MT1** **Ctrl**

**Supplementary Figure 6. Correlation of the findings related to severity and control group (T1).** Correlation matrix of the significant findings presented for the moderately ill patients at T1 and for the healthy controls for all outcomes (MT1 = moderate at acute infection, T1, n = 12, Ctrl = healthy controls n = 15).
